# Synthesis of *O*^6^-alkylated preQ_1_ derivatives

**DOI:** 10.3762/bjoc.17.147

**Published:** 2021-09-02

**Authors:** Laurin Flemmich, Sarah Moreno, Ronald Micura

**Affiliations:** 1 Institute of Organic Chemistry, Center for molecular Biosciences Innsbruck (CMBI), Innrain 80–82, 6020 Innsbruck, Austria

**Keywords:** deazapurines, heterocycles, pyrrolopyrimidines, queuosine, RNA cofactors, RNA methylation

## Abstract

A naturally occurring riboswitch can utilize 7-aminomethyl-*O*^6^-methyl-7-deazaguanine (m^6^preQ_1_) as cofactor for methyl group transfer resulting in cytosine methylation. This recently discovered riboswitch-ribozyme activity opens new avenues for the development of RNA labeling tools based on tailored *O*^6^-alkylated preQ_1_ derivatives. Here, we report a robust synthesis for this class of pyrrolo[2,3-*d*]pyrimidines starting from readily accessible *N*^2^-pivaloyl-protected 6-chloro-7-cyano-7-deazaguanine. Substitution of the 6-chloro atom with the alcoholate of interest proceeds straightforward. The transformation of the 7-cyano substituent into the required aminomethyl group turned out to be challenging and was solved by a hydration reaction sequence on a well-soluble dimethoxytritylated precursor via in situ oxime formation. The synthetic path now provides a solid foundation to access *O*^6^-alkylated 7-aminomethyl-7-deazaguanines for the development of RNA labeling tools based on the preQ_1_ class-I riboswitch scaffold.

## Introduction

Methylated preQ_1_ has attracted much attention recently because this compound has been found to function as cofactor for the conserved fold of a non-coding RNA, namely the preQ_1_ class-I riboswitch [1]. This riboswitch acts as a ribozyme by using 7-aminomethyl*-O*^6^-methyl-7-deazaguanine (m^6^preQ_1_) as methyl group donor; it catalyzes self-methylation of a specific cytidine in the aptamer binding pocket, yielding *N*3-methyl cytidine (m^3^C) under release of 7-aminomethyl*-*7-deazaguanine (preQ_1_) [1]. Thus far, present-day riboswitches have only been known to bind – but not to be able to react – with their ligands [2–3]. This new finding now opens exciting avenues for the development of RNA labeling tools [4], in particular, for RNA methylation, and more generally, for RNA alkylation. To this end, robust synthetic routes towards *O*^6^-alkylated 7-aminomethyl-7-deazaguanines are urgently needed and reported here.

## Results and Discussion

### Biological and synthetic background

#### Role of preQ_1_ in queuosine biosynthesis and gene regulation

**Scheme 1:**
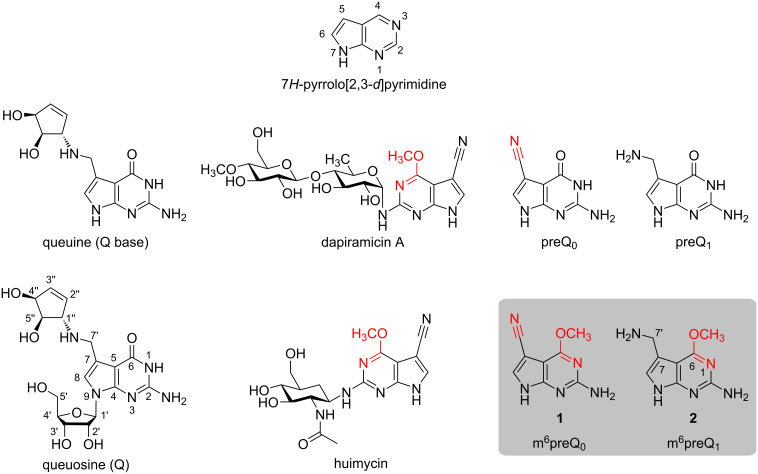
Chemical structures of queuine (Q base) and the hypermodified nucleoside queuosine (Q), the natural products dapiramicin A and huimycin, as well as intermediates of queuosine biosynthesis (preQ_1_ and preQ_0_), and the major synthetic targets of this study, m^6^preQ_0_ (**1**) and m^6^preQ_1_ (**2**) (grey box).

Queuine (Q base) is a derivative of guanine that is involved in the biosynthetic pathway of the hypermodified tRNA nucleoside queuosine (Q) (Scheme 1) [5]. The core structure of the nucleobase is 7-aminomethyl-7-deazaguanine, a pyrrolo[2,3-*d*]pyrimidine also termed prequeuosine base (preQ_1_) [6–7]. In many bacteria, preQ_1_ binds to specific mRNA domains and thereby regulates genes that are required for queuosine biosynthesis [8–16]. The molecular mechanism behind is called riboswitching. For most riboswitches, ligand binding induces a structural change in the untranslated leader sequence of mRNA by formation (or disruption) of a terminator stem (transcriptional control) or repressor stem (translational control). This conformational event signals on or off to gene expression and represents a feedback-type mechanism that is dependent on cellular ligand concentration [13].

#### Natural occurrence of alkylated prequeuosines

Evidence for the natural occurrence of methylated prequeuosine bases stems from a recent study that demonstrated that m^6^preQ_0_ is produced by *Streptomyces* [17]. Moreover, the natural products huimycin [18] and dapiramicin contain m^6^preQ_0_ as core with their 2-NH_2_ group linked to a 2'-acetamido-2'-deoxy-ß-ᴅ-glucopyranosyl residue in huimycin and to a 2-[4'-(4''-*O*-methyl-ß-ᴅ-glucopyranosyl)-6'-deoxy-α-ᴅ-glucopyranosyl] moiety in dapiramicin A [19–20]. In the biosynthetic pathway, the conversion of preQ_0_ into huimycin requires methylation of preQ_0_ and attachment of the *N*-acetylglucosamine moiety as final steps [18]. The methylation reaction is likely to be catalyzed by the product of the gene *huiC*, which encodes a SAM-dependent methyltransferase [18].

To the best of our knowledge, in contrast to m^6^preQ_0_ [17] the reduced counterpart 7-aminomethyl-*O*^6^-methyl-7-deazaguanine m^6^preQ_1_ has not yet been reported to be isolated from natural sources.

#### Earlier syntheses of preQ_1_, preQ_0_ and m^6^preQ_0_

The synthesis of preQ_1_ has been first described by Goto starting from 2-methylthio-6-methoxy-7-methyl-7-deazapurine and requiring more than ten steps [21]. More efficient was a procedure reported by Nishimura applying a Mannich reaction with dibenzylamine–formaldehyde and 2-acylaminopyrrolo[2,3-*d*]pyrimidin-4(3*H*)-one as key step, thereby selectively installing a dibenzylaminomethyl moiety [22]. Exchange of the dibenzylamine group in the Mannich base with NH_3_ provided preQ_1_ [22]. Alternatively, Klebe demonstrated a Michael addition of 2,6-diaminopyrimidin-4-one to the nitroolefin 2-[(2*E*)-3-nitroprop-2-en-1-yl]-1*H*-isoindole-1,3(2*H*)-dione [23]. Finally, Carell reported a cycloaddition route relying on α-brominated 3-phthalimidopropanal and diaminopyrimidin-4-one [24–25]. We further optimized this path for the synthesis of ^15^N-labeled prequeuosine nucleobase derivatives [26] required for advanced NMR spectroscopic applications [27], and for the syntheses of azido- or amino-functionalized preQ_1_ derivatives needed for cellular applications with engineered riboswitches [28]. Finally, we point out that only a single synthetic route has been published to a potential *O*^6^-methylated precursor of m^6^preQ_1_, namely *N*9-trimethysilylethyl protected m^6^preQ_0_ [20]. This synthesis, however, is based on methylation using diazomethane resulting in a mixture of *N*1 and *O*^6^ methylated products, and we therefore did not further consider this path. Finally, one route was described for m^6^preQ_0_ **1** [29] which is similar to the first step we developed for the synthesis of m^6^preQ_1_ **2** as outlined below.

### Synthesis of m^6^preQ_1_, e^6^preQ_1_, and bn^6^preQ_1_

**Scheme 2:**
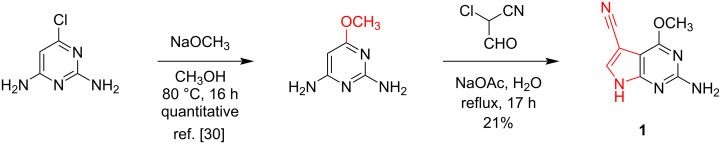
Synthesis of compound **1** (m^6^preQ_0_) by cyclocondensation using a 4-methoxypyrimidine derivative resulted in unsatisfying yields.

Our initial attempts to site-specifically methylate trimethylsilylated preQ_1_ (which was generated in situ with *N*,*O*-bis[trimethylsilyl]acetamide) by trimethyloxonium tetrafluoroborate in apolar solvents resulted in the recovery of starting material only. Next, we tested a cyclocondensation reaction between 2-chloro-3-cyanopropan-1-al and 2,6-diamino-4-methoxypyrimidine [30], however, target compound **1** (m^6^preQ_0_) was obtained in yields of 21% (Scheme 2) which is significantly lower compared to the cyclocondensations with 2,6-diamino-4-pyrimidin-4-one mentioned above [23–27].

**Scheme 3:**
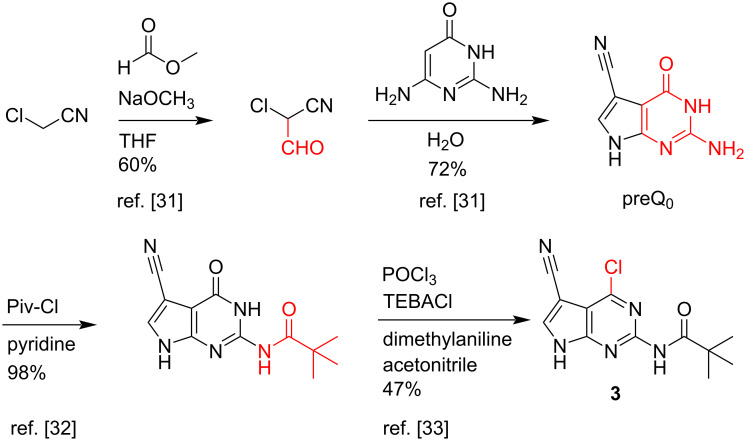
Synthesis of compound **3** following known procedures [31–33].

We therefore envisaged a path involving 6-chloro-7-deazapurine derivative **3** (Scheme 3) as this compound is readily available from cheap starting materials following published procedures. Chloroacetonitrile and methyl formate gave 2-chloro-2-cyanoacetaldehyde which was then reacted with 2,6-diaminopyrimidin-4-one to provide preQ_0_ in good yields [31]. Protection of the exocyclic amino group using pivaloyl chloride was optimized from a published procedure [32] and gave nearly quantitative yields of *N*^2^-pivaloyl preQ_0_ in our hands. Finally, transformation of the 6-carbonyl group by using phosphorus oxychloride gave 6-chloro-7-deazapurine derivative **3** [33]. Notably, attempts to directly transform preQ_0_ (without *N*^2^ protection) into 6-chloro-7-cyano-7-deazaguanine failed in our hands.

**Scheme 4:**
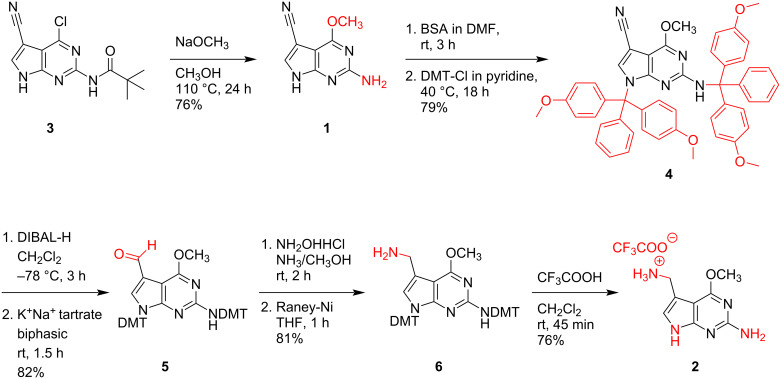
The five-step synthesis of m^6^preQ_1_ **2** from compound **3** required derivatization to make the intermediates soluble in organic solvents for a controllable reaction sequence to reduce the cyano group; overall yield: 30%.

The 6-chloro atom of compound **3** was substituted using sodium methoxide under concomitant cleavage of the pivaloyl group to yield the desired *O*^6^-methylated compound **1**, m^6^preQ_0_ (Scheme 4). After dissolving this compound under strong silylating conditions in the presence of *N*,*O*-bis(trimethylsilyl)acetamide [34], simultaneous tritylation of N9 and the N^2^ atoms was achieved using 4,4'-dimethoxytrityl chloride in pyridine. The obtained derivative **4** was amenable to nitrile reduction using diisobutylaluminium hydride (DIBAL-H) in dichloromethane at −78 °C, followed by workup with potassium sodium tartrate solution (Rochelle salt) to furnish the aldehyde **5**. Then, transformation of the 7-formyl into the 7-aminomethyl group proceeded via oxime formation, applying hydroxylamine hydrochloride in methanolic ammonia, followed by reduction with Raney nickel to yield the tritylated precursor **6**. Finally, the auxiliary functions were cleaved using trifluoroacetic acid (TFA) in dichloromethane and the target compound **2**, m^6^preQ_1_, was isolated as TFA salt.

**Scheme 5:**
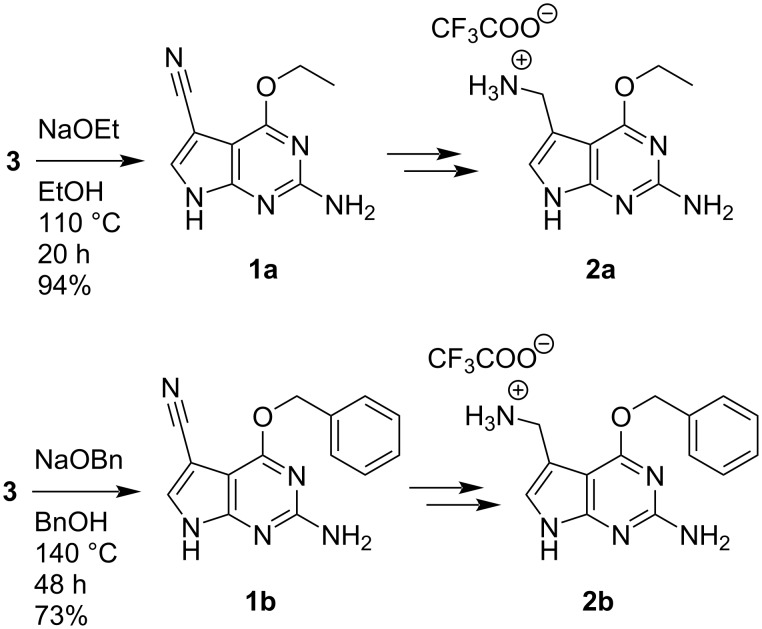
The synthesis of e^6^preQ_1_ (**2a**) and bn^6^preQ_1_ (**2b**) was performed in analogy to the route outlined in Scheme 4 with an overall yield of 23% and 34%, respectively. For details see the Supporting Information File 1.

The here established path to synthesize m^6^preQ_1_ offers high flexibility with respect to the *O*^6^ substituent. We demonstrate this by complementing the set of preQ_1_-derived alkylating cofactors with e^6^preQ_1_ (**2a**) and bn^6^preQ_1_ (**2b**) that were synthesized following the same path with overall yields of 23% and 34%, respectively (Scheme 5 and Supporting Information File 1).

## Conclusion

We developed a robust synthesis for *O*^6^-alkylated 7-aminomethyl-7-deazaguanines that starts from a readily accessible 6-chloro-7-cyano-7-deazaguanine derivative. The chloro atom is smoothly substituted by the alcoholate of interest, followed by N9 and *N*^2^ dimethoxytritylation to obtain a well-soluble intermediate. The reduction of the 7-cyano substituent into the target aminomethyl group is accomplished by a hydration reaction sequence via in situ oxime formation. The route now provides a solid basis to generate *O*^6^-alkylated preQ_1_ derivatives for the development of RNA labeling tools utilizing the RNA scaffold of the preQ_1_ class-I riboswitch.

## Experimental

**General**. Chemical reagents and solvents were purchased from commercial suppliers (Sigma-Aldrich) and used without further purification. Organic solvents for reactions were dried overnight over freshly activated molecular sieves (4 Å). The reactions were carried out under argon atmosphere. Analytical thin-layer chromatography (TLC) was performed on Marchery-Nagel Polygram SIL G/UV254 plates. Column chromatography was done on silica gel 60 (70–230 mesh). ^1^H, and ^13^C NMR spectra were recorded on Bruker DRX 300 MHz, Bruker Avance 4 Neo 400 MHz, and Bruker Avance 4 Neo 700 MHz instruments. Chemical shifts (δ) are reported relative to tetramethylsilane (TMS) and referenced to the residual proton or carbon signal of the deuterated solvent: CDCl_3_ (7.26 ppm) or DMSO-*d*_6_ (2.49 ppm) for ^1^H NMR; CDCl_3_ (77.0 ppm) or DMSO-*d*_6_ (39.5 ppm) for ^13^C NMR spectra. ^1^H and ^13^C assignments are based on COSY, HSQC, and HMBC experiments. MS experiments were performed on a Thermo Fisher QExactive Classic. Samples were analyzed in the positive-ion mode.

***O*^6^-Methyl preQ_0_ (1)** (m^6^preQ_0_)*.* Procedure A: 2-Chloro-3-oxoproanenitrile [29] (6.80 g, 65.6 mmol) was added to a solution of sodium acetate (10.77 g, 131.3 mmol) and 6-methoxypyrimidine-2,4-diamine [33] (9.20 g, 65.6 mmol) in water (270 mL) at 50 °C. After 16 hours, the solution was refluxed for an additional hour and allowed to cool to room temperature. Filtration gave 2.61 g of compound **1** (21%) as grey solid. Procedure B: Sodium (42 mg, 1.8 mmol) was dissolved in 0.8 mL methanol at 0 °C. Compound **3** [29–31] (200 mg, 0.720 mmol) was added and the mixture was heated to 110 °C in a pressure tube for 24 h. After neutralization with glacial acetic acid the volatiles were removed in vacuo. The crude product was dry-loaded onto silica gel and purified via flash column chromatography (5–20% methanol in dichloromethane) to give 104 mg of compound **1** (76%) as a beige solid. TLC: 10% methanol in dichloromethane, *R*_f_ 0.37; ^1^H NMR (300 MHz, DMSO-*d*_6_) δ 12.08 (bs, 1H, HN(9)), 7.81 (s, 1H, HC(8)), 6.45 (s, 2H, H_2_N(2)), 3.96 (s, 3H, H_3_CO(6)) ppm; ^13^C NMR (75 MHz, DMSO-*d*_6_) δ 162.7 (C(6)), 160.7 (C(2)), 154.9 (C(4)), 130.3 (C(8)), 116.1 & 95.4 & 82.4 (C(5) & C(7) & CN), 53.26 (H_3_CO(6)); ESIMS (*m*/*z*): [M + H]^+^ calcd, 190.0723; found, 190.0721.

***N*^2^,9-Bis(4,4'-dimethoxytrityl)-*O*^6^-methyl preQ_0_ (4)**. Compound **1** (300 mg, 1.59 mmol) was suspended in *N,N*-dimethylformamide (12 mL). *N,O*-Bis(trimethylsilyl)acetamide (820 µL, 3.33 mmol) was added dropwise and the reaction mixture was stirred for three hours at room temperature upon which a solution was obtained. Afterwards, the volatile components were removed under reduced pressure and the residue was coevaporated three times with toluene and twice with pyridine. The residue was dissolved in pyridine (3.8 mL) and 4,4'-dimethoxytrityl chloride (1.18 g, 3.50 mmol) was added in portions. The solution was stirred for 18 h at 40 °C, subsequently poured into 5% aqueous sodium bicarbonate solution and the suspension was extracted three times with dichloromethane. The combined organic layers were washed with brine and dried over magnesium sulfate. The solvents were removed and the remaining crude product was purified by flash column chromatography on silica gel (10–30% ethyl acetate in cyclohexane) to give 1.00 g of compound **4** (79%) as a white foam. TLC: 40% ethyl acetate in cyclohexane, *R*_f_ = 0.68; ^1^H NMR (300 MHz, CDCl_3_) δ 7.32–6.94 (m, 19H, HC(aromatic, DMTr) & HC(8)), 6.83–6.65 (m, 8H, HC(arom, DMTr)), 5.54 (s, 1H, HN(2)), 3.80 & 3.77 (s, 12H, H_3_CO(DMTr), 3.37 (s, 1H, H_3_CO(O6) ppm; ^13^C NMR (101 MHz, CDCl_3_) δ 162.4 (C(6)), 158.7 & 158.4 & 158.0 (C(aromatic, DMTr)), 154.8 (C(4)), 146.3 (C(2)), 142.15 & 138.4 & 134.3 (C(aromatic, DMTr)), 133.1 (C(8)), 131.4 & 130.4 & 130.3 & 130.2 & 129.80 & 129.1 & 127.8 & 127.5 & 127.4 & 126.4 (C(aromatic, DMTr)), 115.8 (C(5)/C(7)), 113.3 & 113.1 & 112.7 (C(aromatic, DMTr)), 99.2 (C(5)/C(7)), 83.1 (CN(nitrile)), 76.1 & 70.5 (CAr_3_(DMTr)), 55.4 & 55.3 (H_3_CO(DMTr)), 53.88 (H_3_CO(6)) ppm; ESIMS (*m*/*z*): [M + H]^+^ calcd, 794.3337; found, 794.3321.

**7-Formyl-*N*^2^,9-bis(4,4'-dimethoxytrityl)-*O*^6^-methyl-7-deazaguanine (5)**. To a cooled solution (–78 °C) of compound **4** (1.00 g, 1.26 mmol) in dichloromethane (8 mL) diisobutylaluminium hydride (1 M in dichloromethane, 1.6 mL, 2.57 mmol) was added dropwise. The reaction was continued for three hours, quenched by the addition of ethyl acetate (4 mL) and allowed to come to room temperature. Half-saturated potassium sodium tartrate solution was added (4 mL) and the biphasic mixture was stirred vigorously for one and a half hour until satisfactory phase separation was achieved. The aqueous layer was separated and subsequently extracted three times with ethyl acetate. The combined organic layers were washed with brine, dried over magnesium sulfate and evaporated. The residue was purified by flash column chromatography on silica gel (10–25 % ethyl acetate in cyclohexane) to give 823 mg of compound **5** (82%) as a white foam. TLC: 30% ethyl acetate in cyclohexane *R*_f_ 0.51; ^1^H NMR (CDCl_3_, 400 MHz) δ 9.91 (s, 1H, CHO), 7.49 (s, 1H, HC(8)), 7.29–7.23 (m, 2H, HC(aromatic, DMTr), 7.20–7.11 (m, 6H, HC(aromatic, DMTr), 7.10–7.06 (m, 2H, HC(aromatic, DMTr)), 7.05–6.92 (m, 8H, HC(aromatic, DMTr)), 6.81–6.76 (m, 4H, HC(aromatic, DMTr)), 6.72–6.68 (m, 4H, HC(aromatic, DMTr)), 5.51 (s, 1H, HN(2)), 3.80 (s, 6H, H_3_CO(DMTr)), 3.77 (s, 6H, H_3_CO(DMTr)), 3.38 (s, 3H, H_3_CO(6)) ppm; ^13^C NMR (CDCl_3_, 101 MHz) δ 185.7 (CHO), 163.2 (C(6)), 158.7 & 158.0 & 156.5 (C(aromatic, DMTr)), 156.5 (C(4)), 146.4 (C(2)), 142.3 & 138.6 & 134.5 (C(aromatic, DMTr)), 131.9 (C(8)), 131.5 & 130.3 & 129.9 & 129.1 & 127.7 & 127.4 & 127.3 & 126.4 (C(aromatic, DMTr)), 115.7 (C(5)/C(7)), 113.1 & 112.7 (C(aromatic, DMTr)), 98.0 (C(5))/C(7)), 76.0 (CAr_3_ (DMTr)), 70.5 (DMTr), 55.4 & 33.3 (H_3_CO(DMTr)), 54.0 (H_3_CO(6)) ppm; ESIMS (*m*/*z*): [M + H]^+^ calcd, 797.3334; found, 797.3315.

**7-Aminomethyl-*N*^2^,9-bis(4,4'-dimethoxytrityl)-*O*^6^-methyl-7-deazaguanine (6)**. To a suspension of compound **5** (200 mg, 0.251 mmol) in 7 M methanolic ammonia (6 mL) hydroxylamine hydrochloride (21 mg, 0.300 mmol) was added. A clear solution was obtained shortly thereafter. The reaction was stirred for two hours at room temperature. Tetrahydrofuran (4 mL) and damp Raney-Nickel (approximately 200 mg) were introduced and the reaction was continued for one hour. The reaction mixture was filtered over a Celite pad and the filtrate was evaporated. The residue was taken up in 3% methanol in dichloromethane and passed over a short, deactivated silica pad and evaporated once more to give 163 mg of compound **6** (81%) as a white foam. TLC: 6% MeOH, 1% NEt_3_ in dichloromethane, *R*_f_ 0.26; ^1^H NMR (CDCl_3_, 400 MHz) δ 7.24–7.20 (m, 3H, HC(aromatic, DMTr)), 7.18–7.08 (m, 7H, HC(aromatic, DMTr)), 7.06–7.00 (m, 8H, HC(aromatic, DMTr)), 6.77–6.67 (m, 7H, HC(aromatic, DMTr)), 6.44 (s, 1H, HC(8)), 5.46 (s, 1H, HN(2)), 3.79 (s, 6H, H_3_CO(DMTr)), 3.77 (s, 6H, H_3_CO(DMTr)), 3.67 (s, 2H, H_2_CC(7)), 3.33 (s, 3H, H_3_CO(6)), 1.81 (bs, 2H, H_2_N) ppm; ^13^C NMR (CDCl_3_, 101 MHz) δ 162.3 (C(6)), 158.3 & 157.8 & 157.2 & (C(arom, DMTr)), 155.9 (C(4)), 146.8 (C(2)), 143.6 & 139.0 & 135.9 & 131.4 130.4 & 130.0 & 129.2 & 127.4 & 127.3 & 126.8 & 126.2 (C(arom, DMTr)), 121.4 (C(8)), 115.9 (C(5)) or (C(7)), 112.71 & 112.6 (C(arom, DMTr)), 74.5 (CAr_3_(DMTr)), 70.3 (CAr_3_(DMTr)), 55.3 (H_3_CO(DMTr)), 53.4 (H_3_CO(6)), 38.9 (H_2_*C*C(7)) ppm; ESIMS (*m*/*z*): [M + H]^+^ calcd, 798.3650; found, 798.3629.

***O*^6^-Methyl preQ_1_ (trifluoroacetate salt) (2)**. Compound **6** (120 mg, 150 µmol) was dissolved in 500 µL dichloromethane. Trifluoroacetic acid (60 µL, 0.75 mmol) and 10 µL water were added. After 45 minutes the reaction was quenched by the addition of 100 µL methanol. Afterwards the solvents were removed in vacuo. The residue was triturated five times with dichloromethane and dried on high vacuum to give 35 mg of compound **2** (76%) as a white solid. TLC: 15% MeOH, 1% NEt_3_ in dichloromethane, *R*_f_ 0.56; ^1^H NMR (CDCl_3_, 400 MHz) δ 11.64 (bs, 1H, HN(9), 8.07 (bs, 3H, H_3_N^+^), 6.99 (d, 1H, *J*_HH_ = 2.0 Hz, HC(8)), 4.06 (q, 2H, H_2_CC(7)), 3.98 (s, 3H, H_3_CO) ppm; ^13^C NMR (CDCl_3_, 101 MHz) δ 164.1 (C(6)), 158.4 (q, *J*_CF_ = 34.0 Hz, CF_3_*C*OO^−^), 158.1 (C(2)), 150.7 (C(4)), 120.0 (C(8)), 116.3 (q, *J*_CF_ = 295.0 Hz, *C*F_3_COO^−^), 107.5 & 96.2 ((C(5) & C(7)), 53.7 (H_3_CO), 34.8 (CH_2_CC(7)) ppm; ESIMS (*m*/*z*): [M + H – NH_3_]^+^ calcd, 177.0771; found, 177.0767; [M + H]^+^ calcd, 194.1036; found, 194.1032.

## Supporting Information

File 1 Synthetic procedures for compounds **1a**–**6a**, and **1b**–**6b**, and ^1^H and ^13^C NMR spectra of all compounds. ^1^H,^13^C HSQC and ^1^H,^13^C HMBC spectra of all final products **1**, **1a**, **1b**, **2**, **2a**, and **2b**.
