# Supplementary material for: Synthesis of O6-alkylated preQ1 derivatives
Source: Beilstein J Org Chem. 2021 Sep 2;17:2295–301. doi: 10.3762/bjoc.17.147 (PMC8450960; doi:10.3762/bjoc.17.147)

BEILSTEIN JOURNAL OF ORGANIC CHEMISTRY

Supporting Information

for

Synthesis of O^6-alkylated preQ$_1$ derivatives

Laurin Flemmich, Sarah Moreno and Ronald Micura

Beilstein J. Org. Chem. **2021,** *17,* 2295–2301. doi:10.3762/bjoc.17.147

Synthetic procedures for compounds 1a–6a, and 1b–6b, and ^{1}H and ^{13}C NMR spectra of all compounds. ^{1}H,^{13}C HSQC and ^{1}H,^{13}C HMBC spectra of all final products 1, 1a, 1b, 2, 2a, and 2b

License and Terms: This is a supporting information file under the terms of the Creative Commons Attribution License (https://creativecommons.org/licenses/by/4.0). Please note that the reuse, redistribution and reproduction in particular requires that the author(s) and source are credited and that individual graphics may be subject to special legal provisions.
The license is subject to the *Beilstein Journal of Organic Chemistry* terms and conditions: (https://www.beilstein-journals.org/bjoc/terms)

Contents

1. Synthesis of m^6preQ_1 (trifluoroacetate salt) **2** S2

1.1. NMR spectra of compounds **1**, **4**, **5**, **6**, and **2** S3

2. Synthesis of e^6preQ_1 (trifluoroacetate salt) **2a** S10

2.1. *O*[6]-Ethyl $preQ_0$ **1a** S10

2.2. N^2,9-Bis(-4,4'-dimethoxytrityl)-O^6-ethyl $preQ_0$ **4a** S11

2.3. 7-Formyl-N^2,9-bis(4,4'-dimethoxytrityl)-O^6-ethyl-7-deazaguanine **5a** S11

2.4. 7-Aminomethyl-N^2,9-bis(4,4'-dimethoxytrityl)-O^6-ethyl-7-deazaguanine **6a** S12

2.5. O^6-Ethyl $preQ_1$ (trifluoroacetate salt) **2a** S13

2.6. NMR spectra of compounds **1a**, **4a**, **5a**, **6a**, and **2a** S14

3. Synthesis of bn^6preQ_1 (trifluoroacetate salt) **2b** S21

3.1. O^6-Benzyl $preQ_0$ **1b** S21

3.2. N^2,9-Bis-(4,4'-dimethoxytrityl)-O^6-benzyl $preQ_0$ **4b** S22

3.3. 7-Formyl-N^2,9-bis(4,4'-dimethoxytrityl) O^6-benzyl-7-deazaguanine **5b** S22

3.4. 7-Aminomethyl-N^2,9-bis(4,4'-dimethoxytrityl)-O^6-benzyl-7-deazaguanine **6b** S23

3.5. O^6-Benzyl $preQ_1$ (trifluoroacetate salt) **2b** S24

3.6. NMR spectra of compounds **1b**, **4b**, **5b**, **6b**, and **2b** S25

1. Synthesis of m^{6}preQ$_1$ (trifluoroacetate salt) (2)

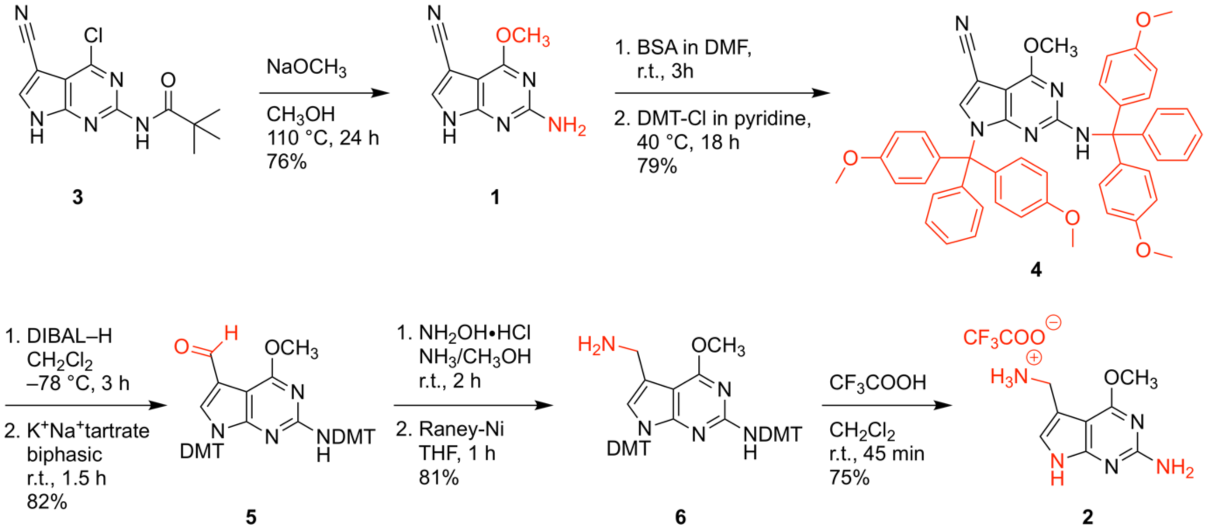

Supporting Scheme 1. Synthetic overview for the trifluoroacetate salt of m^{6}preQ$_1$ **2**.

All synthetic procedures for the transformations shown in Supporting Scheme 1 are described in the main text.

1.1. NMR spectra of compounds 1, 4, 5, 6, and 2

^{1}H-NMR (300 MHz, DMSO-d_6) of compound **1**:

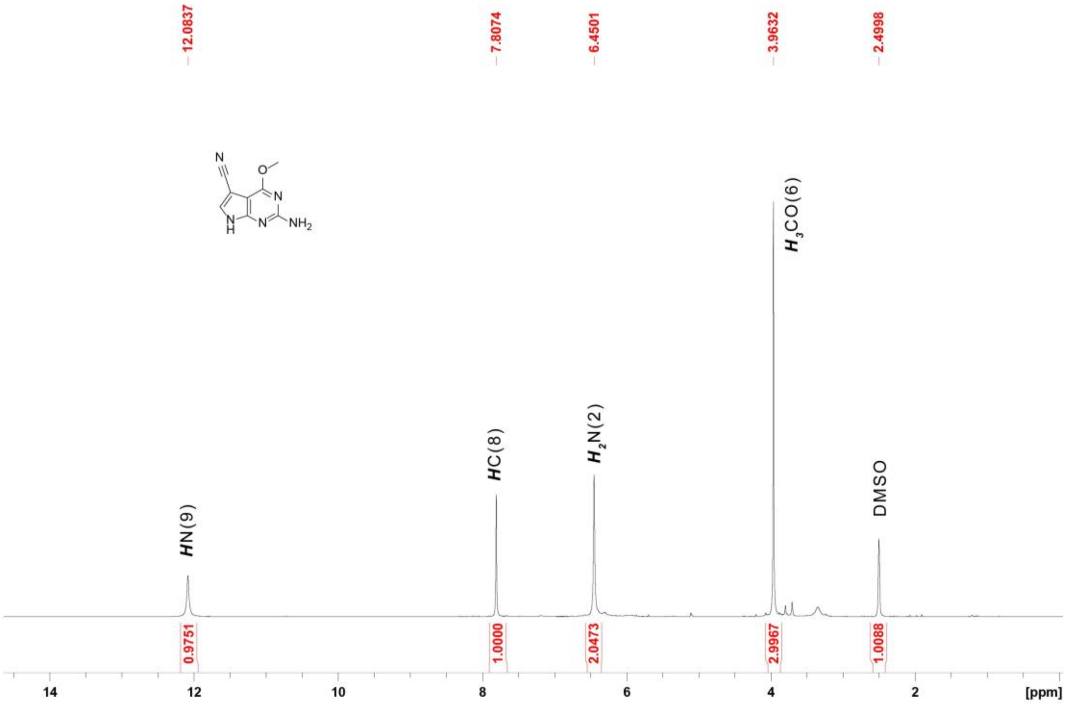

^{13}C-NMR (75 MHz, $CDCl_3$) of compound **1**:

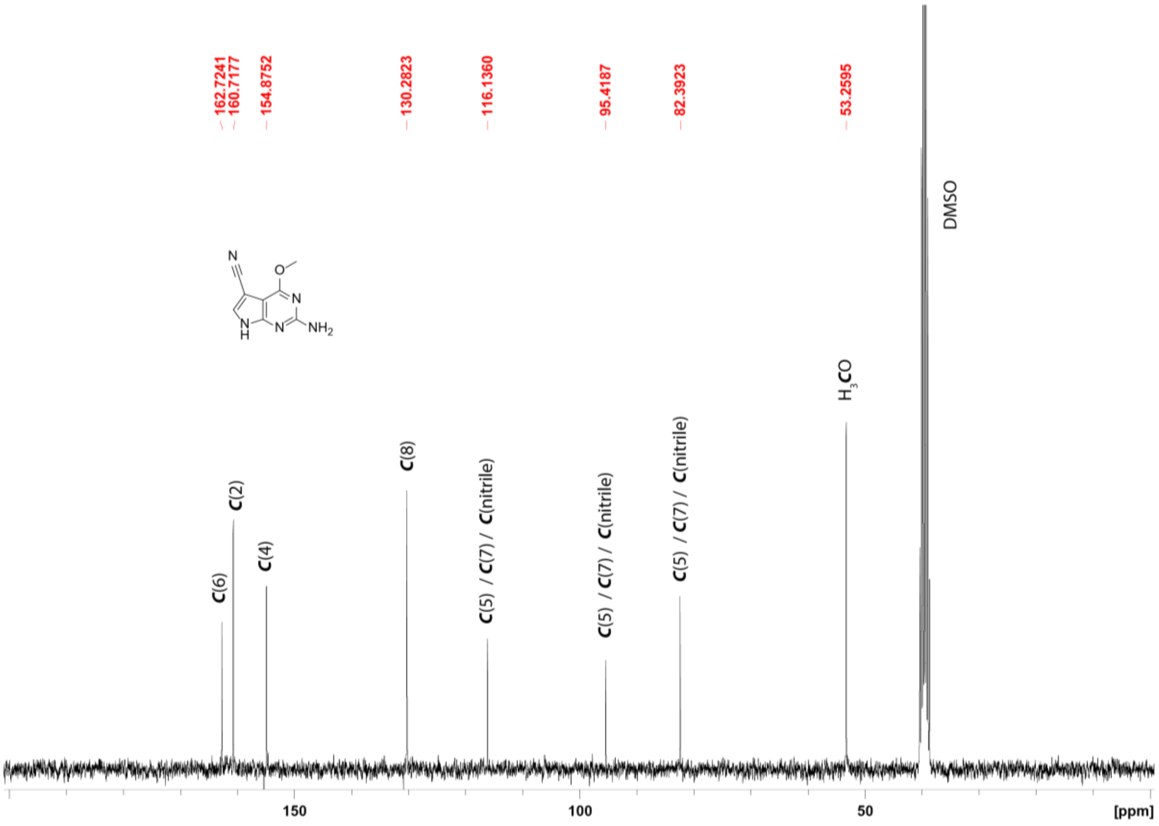

^{1}H-^{13}C HSQC NMR (300 MHz, DMSO-d_6) of compound **1**:

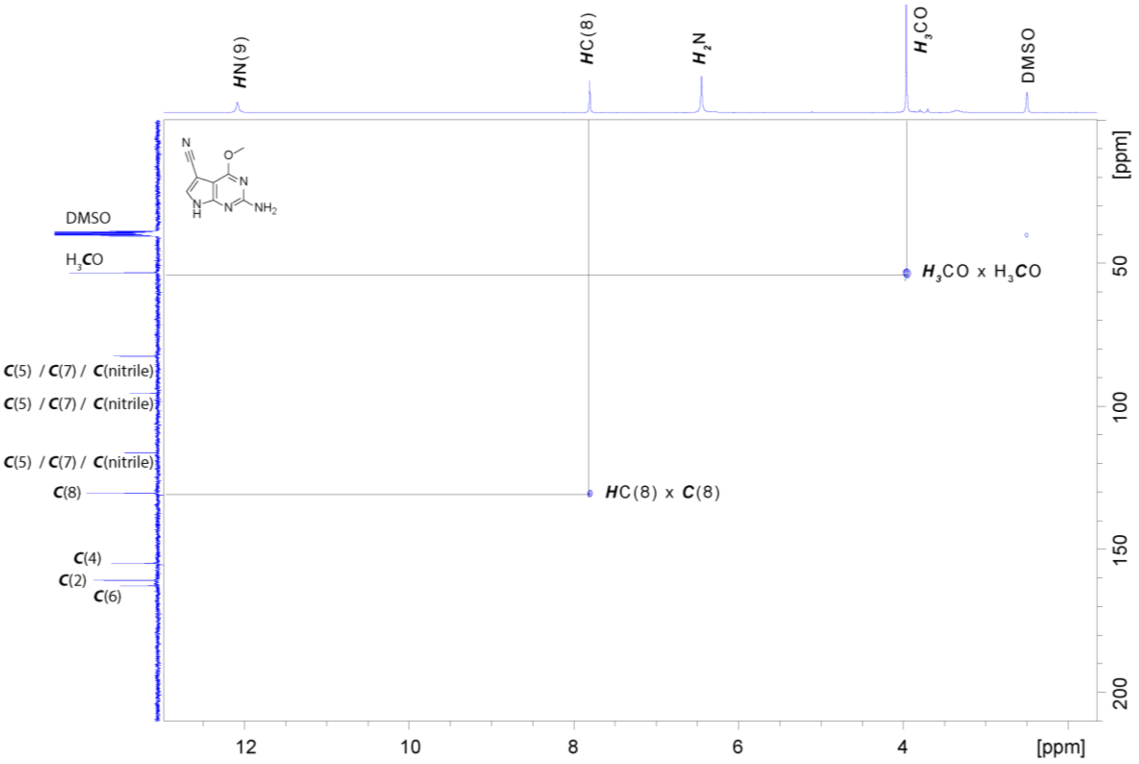

^{1}H-^{13}C HMBC NMR (300 MHz, DMSO-d_6) of compound **1**:

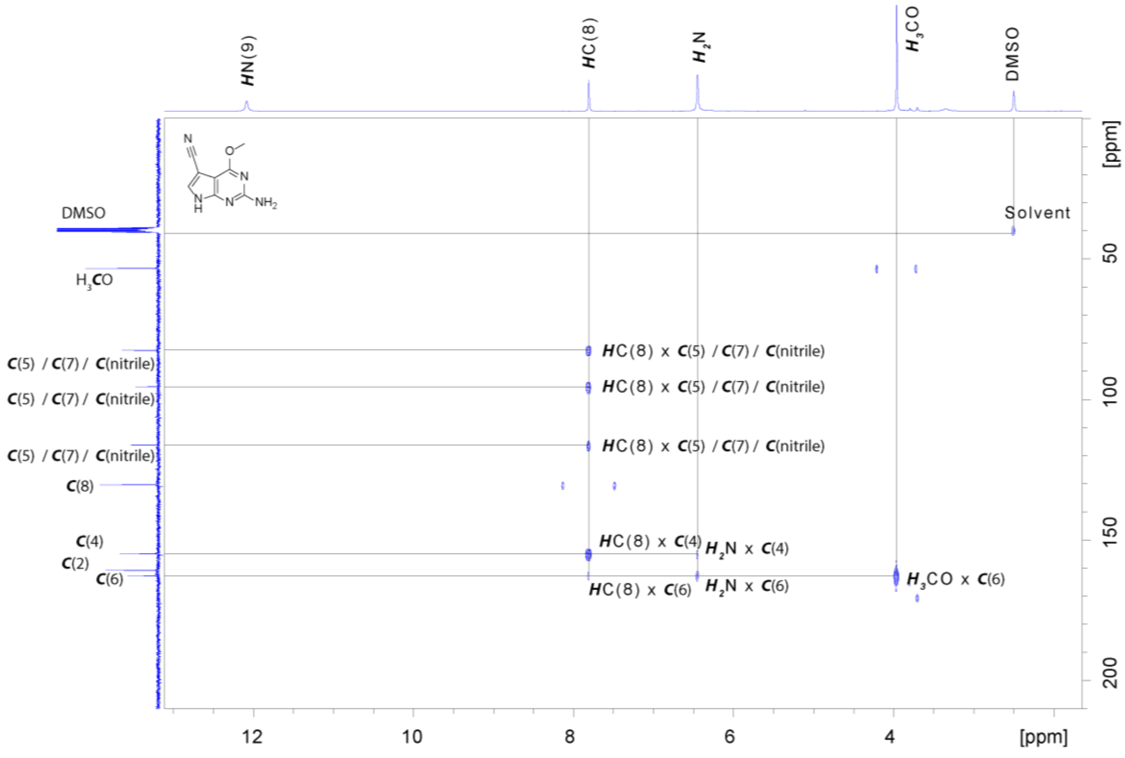

^{1}H-NMR (300 MHz, $CDCl_3$) of compound **4**:

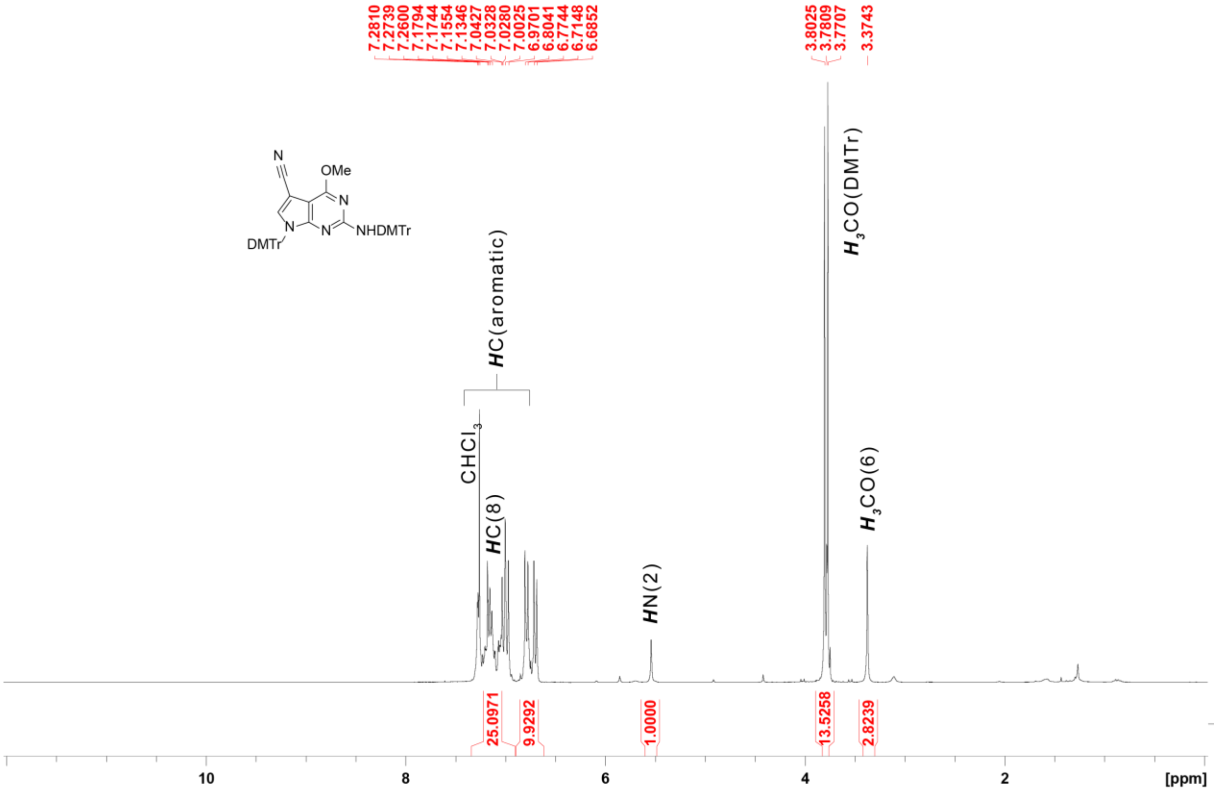

^{13}C-NMR (75 MHz, 300 MHz, $CDCl_3$) of compound **4**:

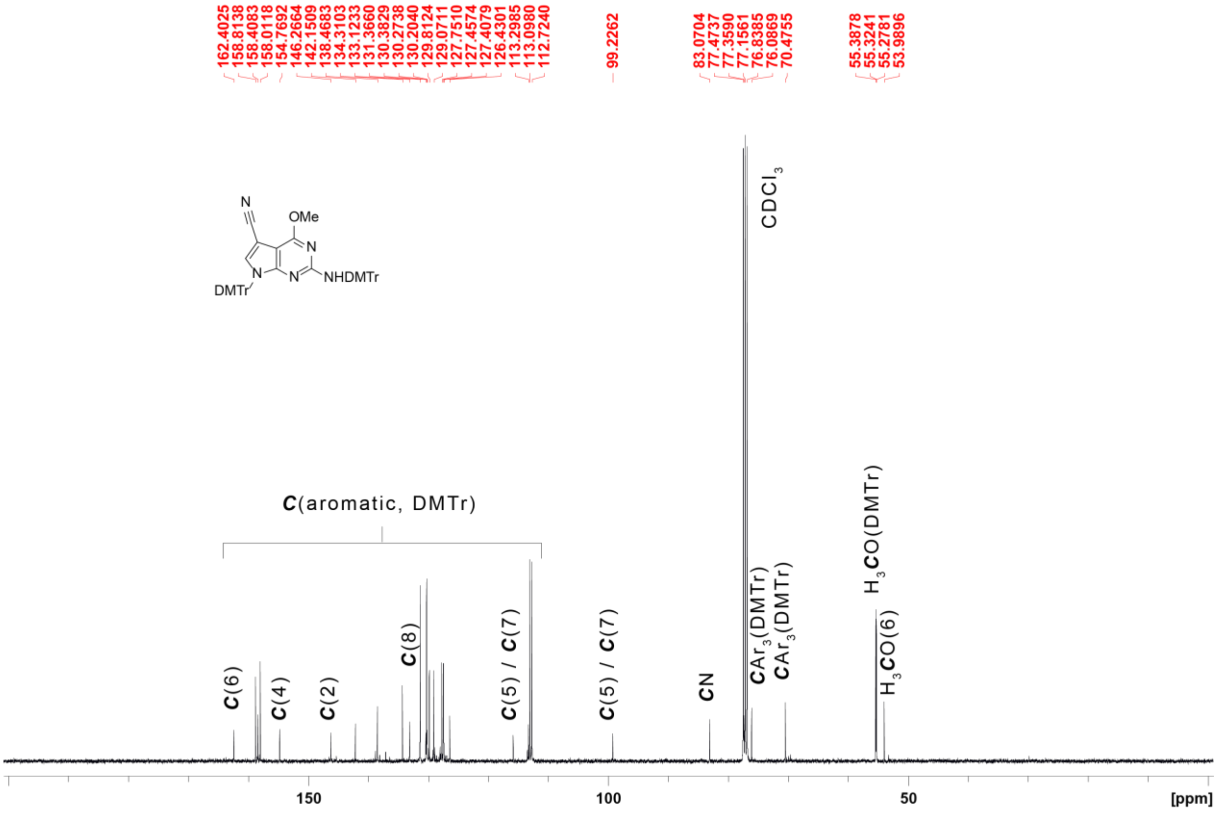

^{1}H-NMR (400 MHz, $CDCl_3$) of compound **5**:

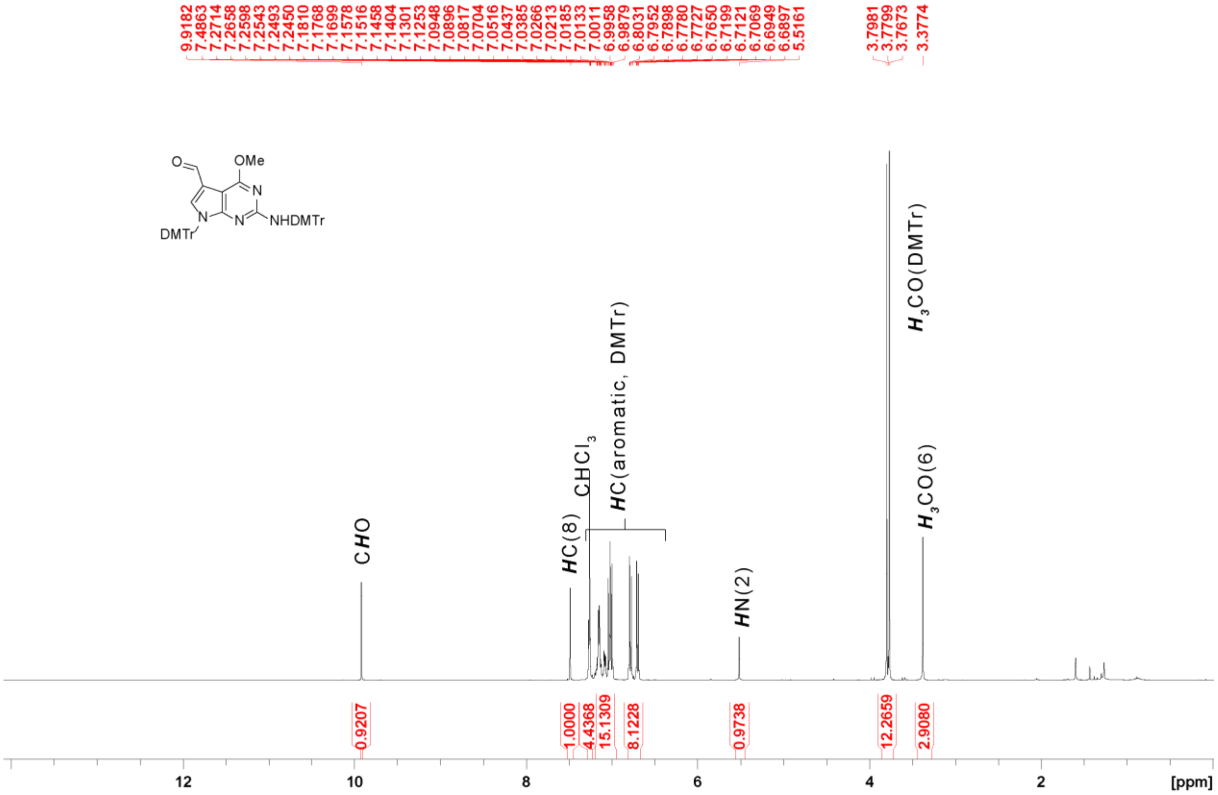

^{13}C-NMR (101 MHz, $CDCl_3$) of compound **5**:

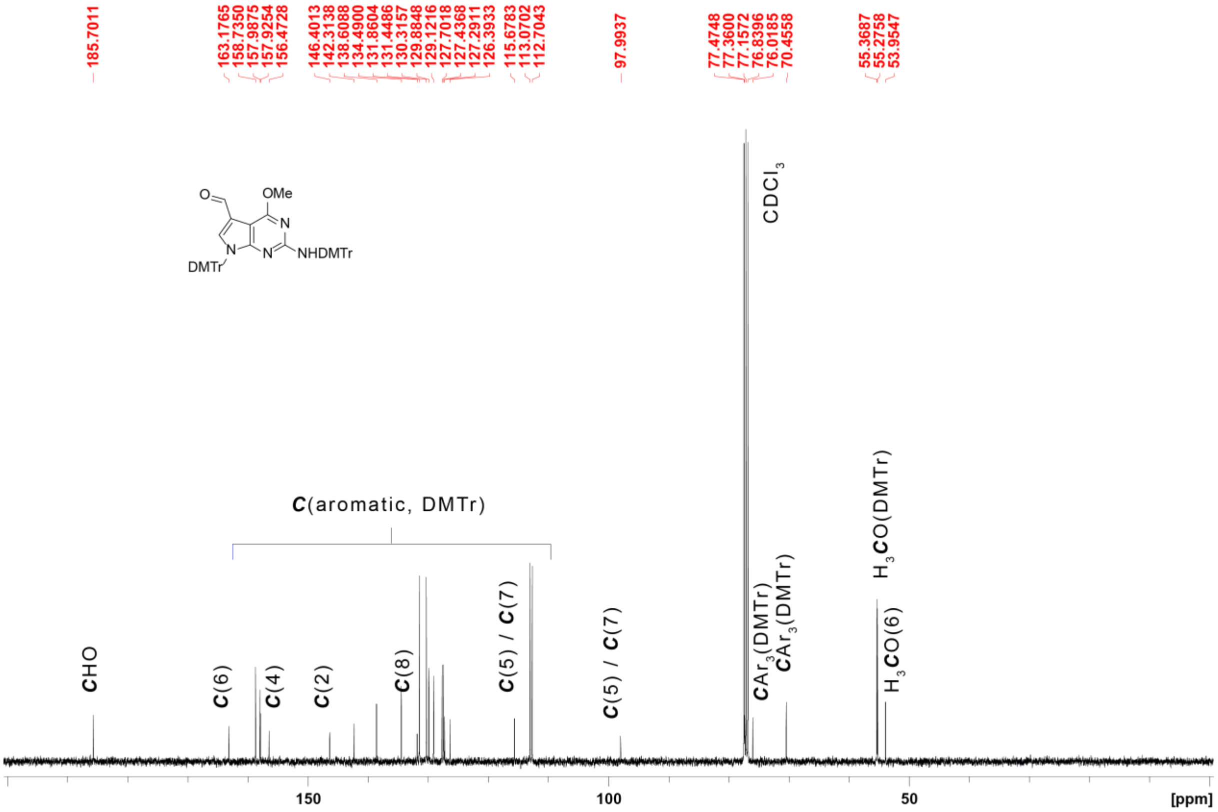

^{1}H-NMR (400 MHz, $CDCl_3$) of compound **6**:

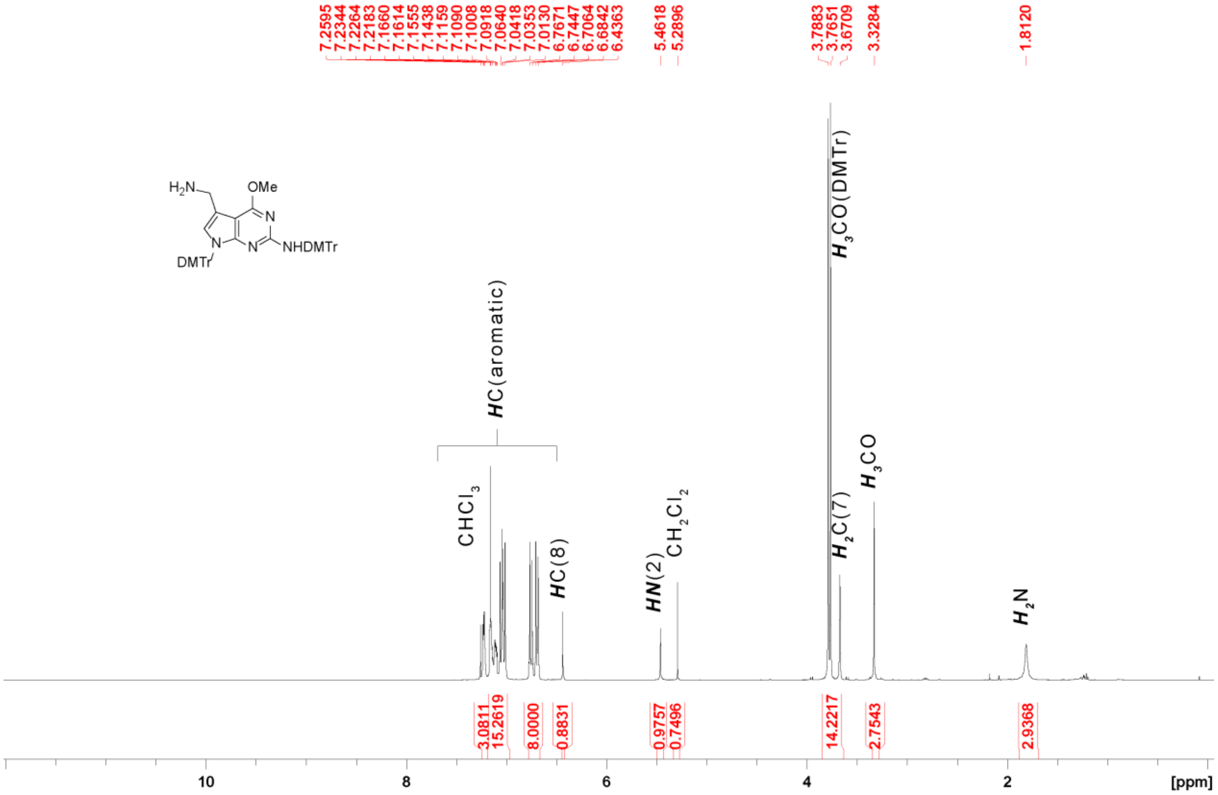

^{13}C-NMR (101 MHz, $CDCl_3$) of compound **6**:

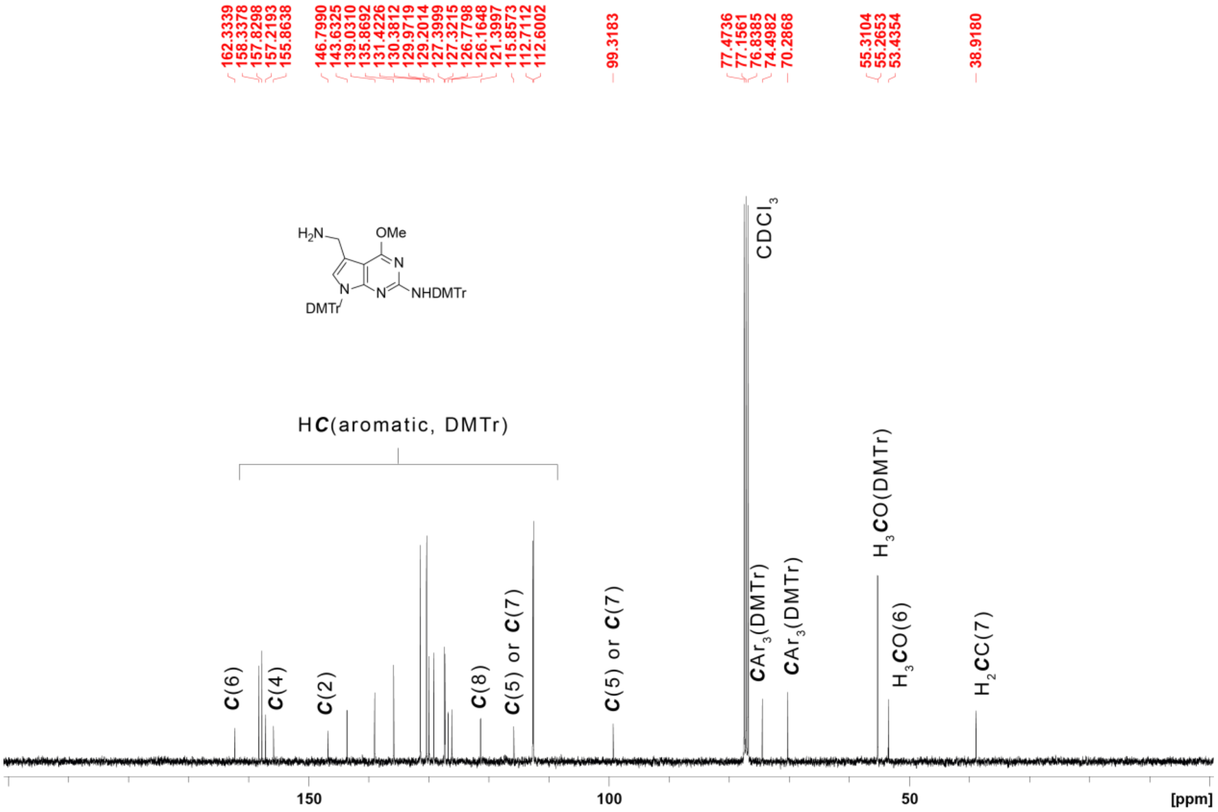

^{1}H-NMR (400 MHz, DMSO-d_6) of compound **2**:

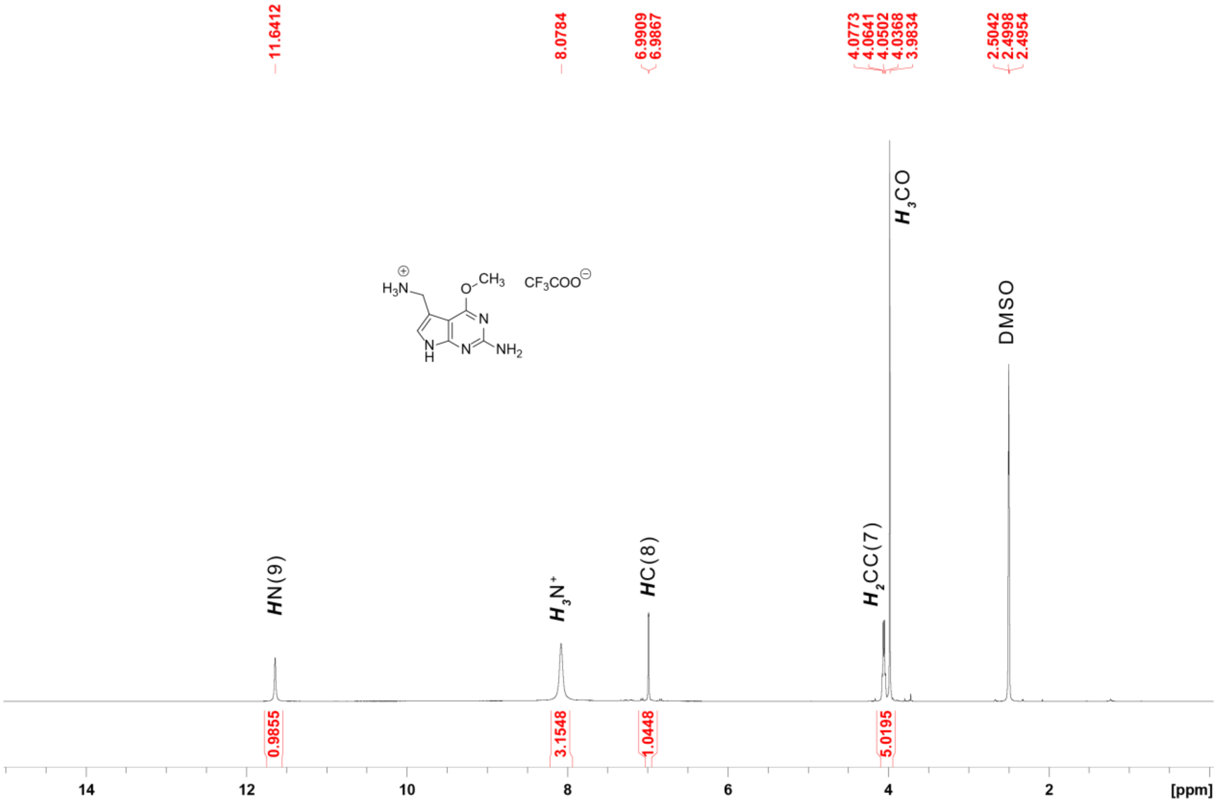

^{13}C-NMR (101 MHz, DMSO-d_6) of compound **2**:

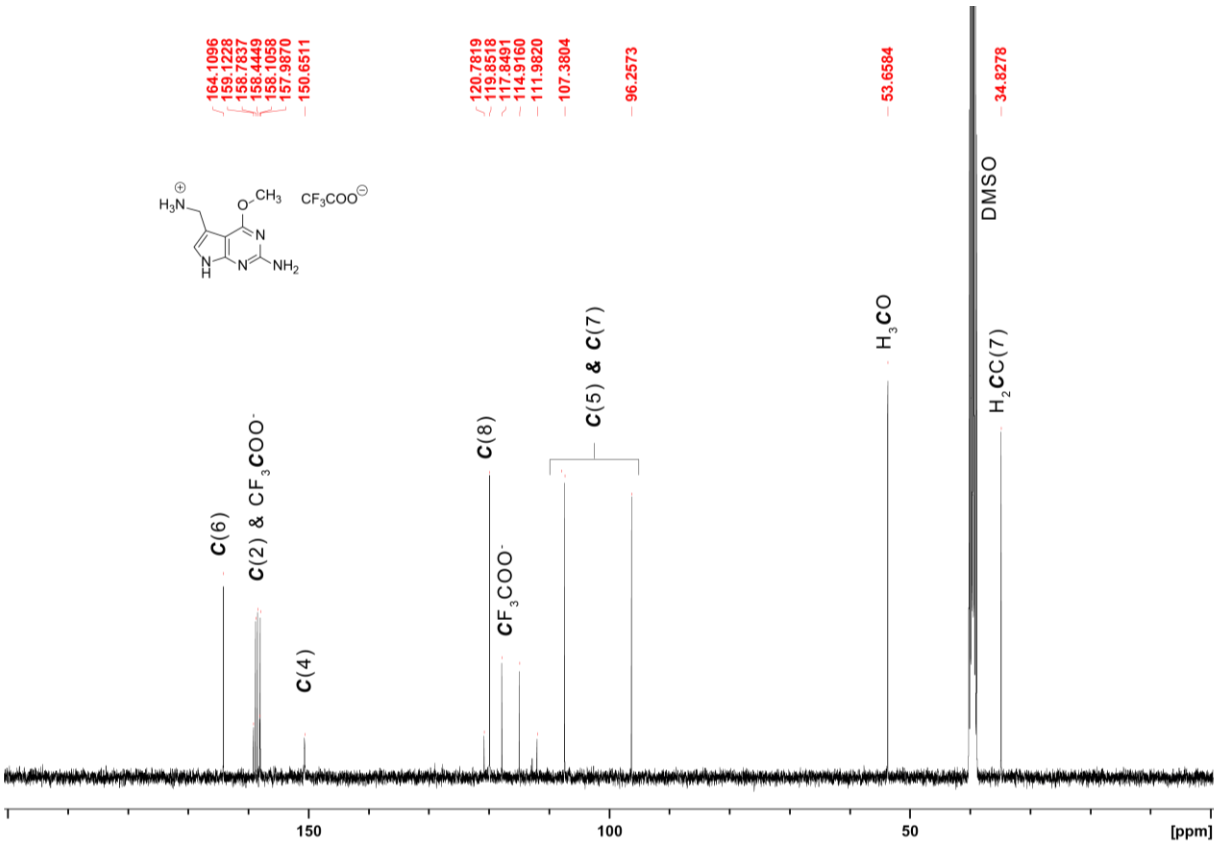

^1H-^{13}C HSQC NMR (300 MHz, DMSO-d_6) of compound **2**:

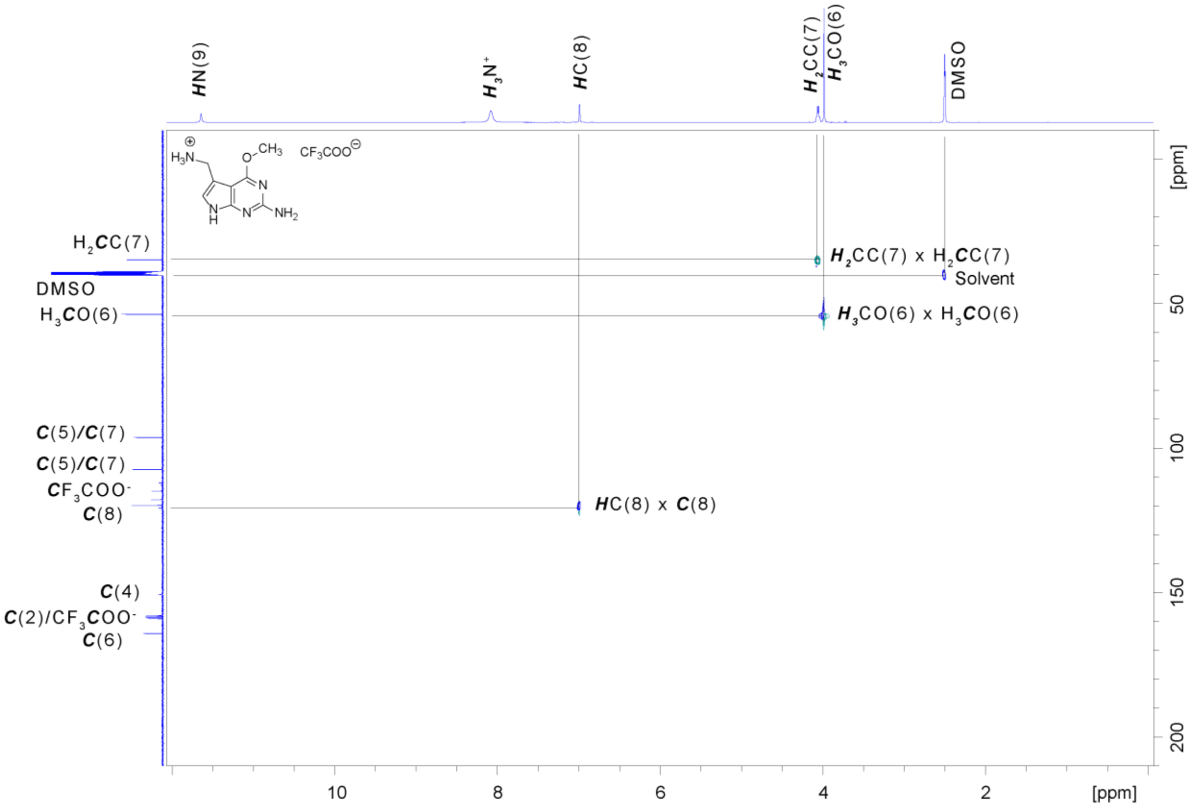

^1H-^{13}C HSQC NMR (300 MHz, DMSO-d_6) of compound **2**:

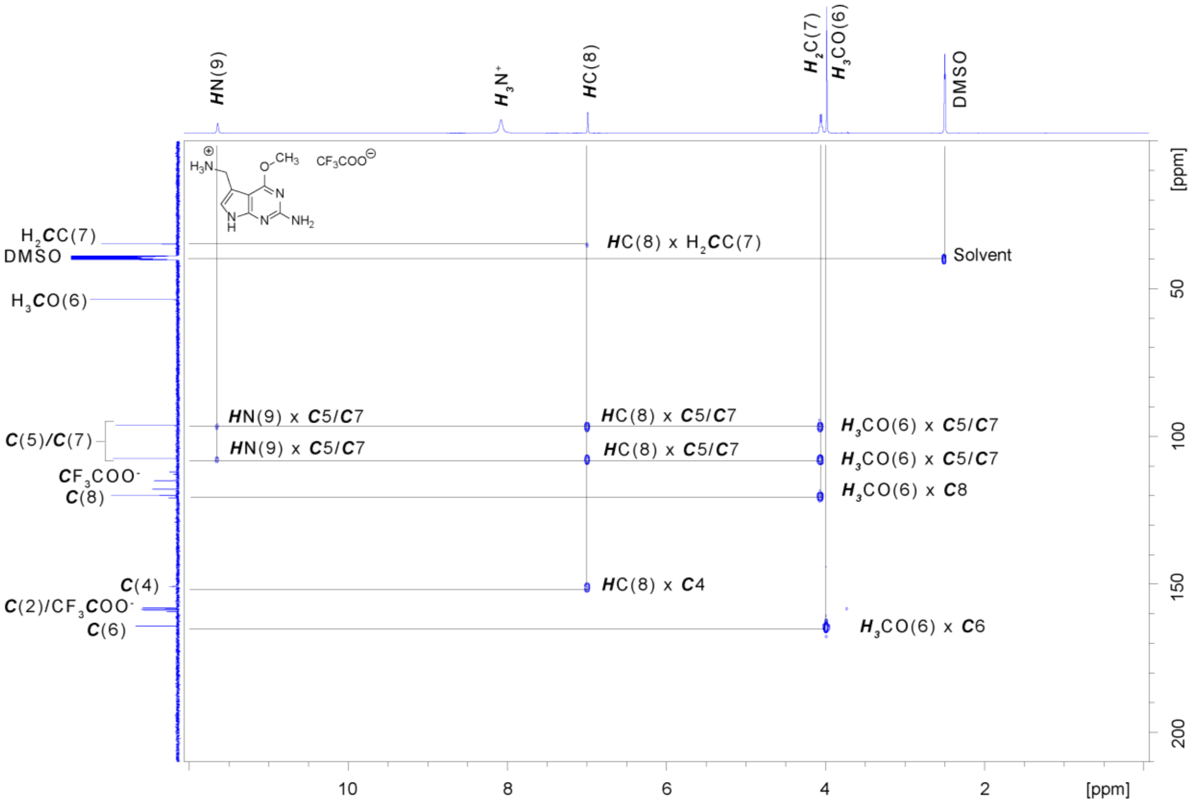

2. Synthesis of e^{6}preQ$_1$ (trifluoroacetate salt) (2a)

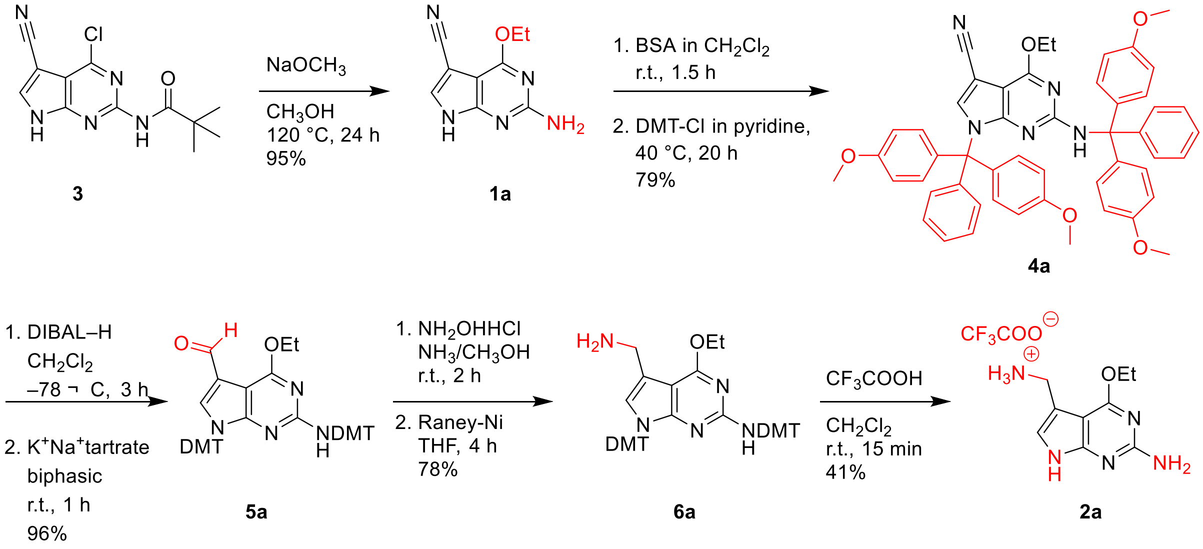

Supporting Scheme 2. Synthetic overview of the trifluoroacetate salt of e^{6}preQ$_1$ **2a**.

2.1. *O*^{6}-Ethyl preQ$_0$ (1a)

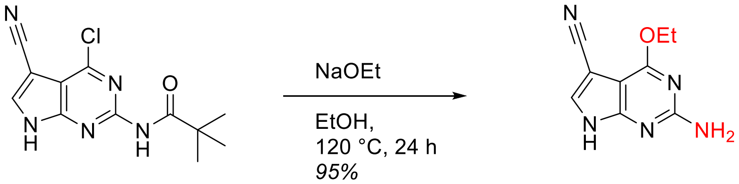

3
$C_{12}H_{12}ClN_5O$
277.71

1a
$C_9H_9N_5O$
203.21

Sodium (2.22 mmol, 51 mg) was dissolved in ethanol (4.0 ml) and compound **3** (0.77 mmol, 213 mg) was added shortly after. The reaction mixture was stirred in a pressure tube for 24 h at 120 °C, was cooled to room temperature and was neutralized by the addition of acetic acid. Solvent was removed in vacuo and the powdery residue was adsorbed on silica. Flash chromatography (100% EtOAc) afforded compound **1a** (148 mg, 95%) as a slightly yellow powder. TLC: 8% methanol in dichloromethane; R_f 0.40

^{1}H-NMR (DMSO-*d*$_6$, 400 MHz): δ 1.35 (t, 3 H, $^3J_{HH}$ = 7.07 Hz, H_3C); 4.44 (q, 2H, $^3J_{HH}$ = 7.06 Hz, $H_2CO(6)$); 6.40 (bs, 2H, $H_2N(2)$); 7.79 (d, 1H, $^3J_{HH}$ = 1.50 Hz, C(8)); 12.04 (bs, 1H, HN(9)) ppm.

^{13}C-NMR (DMSO-*d*$_6$, 101 MHz): δ 14.38 CH_3; 61.39 CH_2; 82.47 C (5) / C (7) / CN, 95.39 C(5) / C(7) / CN(nitrile); 116.03 C(5) / C (7) / CN; 130.16 C(8); 154.88 C(2); 160.73 C(4), 162.38 C(6) ppm.

ESI-MS (m/z): $[M+H]^+$ calculated 204.0880; found: 204.0879.

2.2. *N*^{2},9-Bis(4,4'-dimethoxytrityl)-*O*^{6}-ethyl preQ$_0$ (4a)

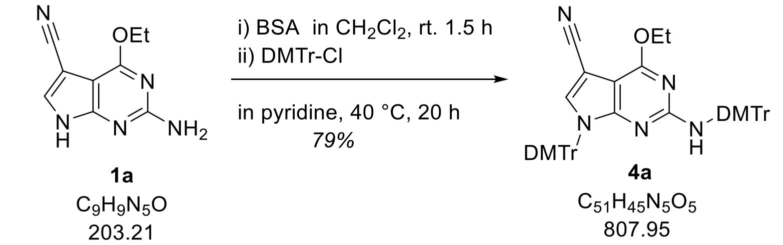

Compound **1a** (128 mg, 0.63 mmol) was dissolved in dichloromethane (2.1 mL) and *N,O*-bis(trimethylsilyl)acetamide (0.32 mL, 1.32 mmol) was added. The suspension turned into a clear solution within a couple of minutes and was stirred for another one and a half hour at ambient temperature. The reaction mixture was concentrated, dissolved in pyridine (1.5 mL), and treated with dry 4,4'-dimethoxytrityl chloride (1.45 mmol, 491 mg) in two portions over a period of 30 minutes at 40 °C. After 20 h at 40 °C the reaction mixture was poured into 5% sodium bicarbonate solution and the aqueous layer was extracted three times with ethyl acetate. The combined organic layers were washed with brine, dried over sodium sulfate and concentrated. The oily residue was coevaporated twice with toluene and was purified via column chromatography on silica gel (10–30% ethyl acetate, 1% triethylamine in cyclohexane) to yield 403 mg of compound **4a** (79%) as a white foam. TLC: 30% ethyl acetate in cyclohexane; R_f 0.55

^{1}H-NMR ($CDCl_3$, 400 MHz): δ 1.05 (t, 3H, $^3J_{HH}$ = 7.13 Hz, H_3C); 3.73-3.80 (m, 2H, $H_2CO(6)$); 3.77 & 3.80 (s, 12H H_3CO(DMT)); 5.51 (bs, 1H, HN(2)); 6.68-6.70 & 6.77-6.79 & 6.97-7.16 & 7.25-7.29 (m, 26H, HC(aromatic, DMTr)), 7.15 (s, 1H, HC(8)) ppm.

^{13}C-NMR ($CDCl_3$, 101 MHz): δ 14.41 CH_3, 55.29 & 55.39 CH_3O(DMTr), 62.34 $CH_2O(6)$; 70.34, 76.04 C (aromatic, DMTr), 83.19 CN(nitrile), 99.21 C(5) / C(7); 112.77 &113.08 (aromatic, DMTr), 115.78 C(5) / C (7); 126.39 & 127.39 & 127.49 & 127.73 & 129.02 & 129.84 & 130.22 & 131.39 (aromatic, DMTr), 133.05 C(8), 134.36 & 138.54 & 142.20 (aromatic, DMTr), 146.29 C(2); 154.78 C(4), 158.00 & 158.47 & 158.80 (aromatic, DMTr), 162.21 C(6) ppm.

ESI-MS (m/z): $[M+H]^+$ calculated 808.3493; found: 808.3475.

2.3. 7-Formyl-*N*^{2},9-bis(4,4'-dimethoxytrityl)-*O*^{6}-ethyl-7-deazaguanine (5a)

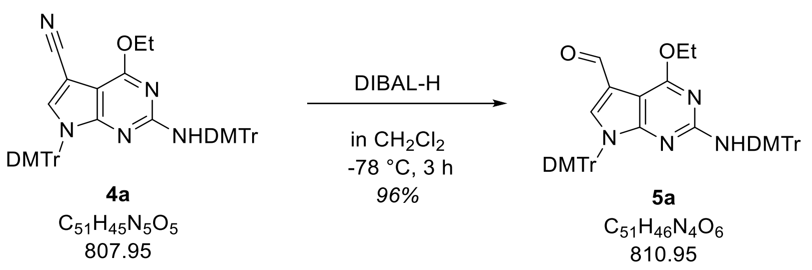

Compound **4a** (395 mg, 0.49 mmol) was dissolved in dichloromethane (3.1 mL) and the solution was cooled to –78 °C. Diisobutylaluminium hydride (0.62 mL, 1 M in CH_2Cl_2, 0.63 mmol) was added dropwise over 30 minutes and stirring was continued for another three hours at –78 °C. The reaction was quenched by the addition of ethyl acetate (2.0 mL) and was subsequently allowed to warm to

ambient temperature. Half-saturated potassium sodium tartrate solution (2.0 mL) was added and the reaction mixture was stirred vigorously for one hour until satisfactory phase separation was achieved. The phases were separated and the aqueous layer was washed three times with ethyl acetate. The combined organic extracts were washed with brine, dried over sodium sulfate and purified via column chromatography on silica gel (10–30% ethyl acetate, 1% triethylamine in cyclohexane) to give 379 mg of compound **5a** as a white foam (96%). TLC: 30% ethyl acetate in cyclohexane, R_f 0.50.

^{1}H-NMR ($CDCl_3$, 400 MHz): δ 1.06 (t, 3H, $^3J_{HH}$ = 7.08 Hz, H_3C), 3.72-3.77 (m, 2H, $H_2CO(6)$), 3.77 & 3.79 (s, 12H, H_3CO(DMTr)); 5.48 (bs, 1H, NH(2)); 6.68-6.71 & 6.76-6.79 & 6.99-7.15 & 7.25-7.26 (m, 26H, HC(aromatic, DMTr), 7.50 (s, 1H, HC(8)); 10.01 (s, 1H, CHO) ppm.

^{13}C-NMR ($CDCl_3$, 101 MHz): δ 14.56 CH_3, 55.28 & 55.35 CH_3O(DMTr); 62.17 $CH_2O(6)$, 70.32 & 75.96 C(aromatic, DMTr), 98.35 C(5) / C(7), 112.73 & 113.05 C(aromatic, DMTr), 115.54 C(5) / C(7); 126.34 & 127.25 & 127.45 & 127.67 & 129.05 & 129.86 & 130.24 C(aromatic, DMTr), 130.60 C(8); 131.44 & 134.51 & 138.67 & 142.34 C(aromatic, DMTr), 146.41 C(2); 156.28 C(4), 157.93 & 157.96 & 158.71 C(aromatic, DMTr), 162.93 C(6), 186.18 CHO ppm.

HR-ESI-MS (m/z): $[M+H]^+$ calculated 811.3490; found: 811.3477

2.4. 7-Aminomethyl-*N*^{2},9-bis(4,4'-dimethoxytrityl)-*O*^{6}-ethyl-7-deazaguanine (6a)

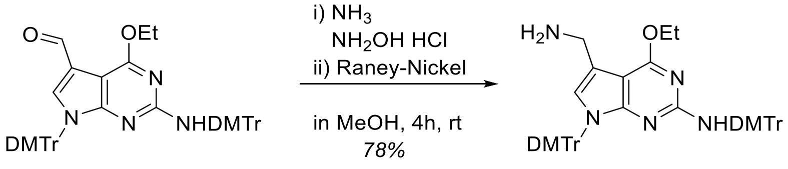

5a
$C_{51}H_{46}N_4O_6$
810.95

6a
$C_{51}H_{49}N_5O_5$
811.98

Hydroxylamine hydrochloride (0.70 mmol, 48 mg) was added to a suspension of compound **5a** (470 mg, 0.58 mmol) in 7 M methanolic ammonia (13.9 mL). The solution was stirred at room temperature for two hours and became colorless. Tetrahydrofuran (9.2 mL) and Raney Nickel (approximately 500 mg) were added and the reaction mixture was stirred under H_2 atmosphere (1 atm, balloon). Additional Raney nickel (approx. 500 mg) was added after three hours. The reaction was deemed complete after 4 h and was then filtered over a pad of Celite. The reaction mixture was concentrated and subjected to column chromatography on silica gel (0-2% MeOH in CH_2Cl_2 + 1% Et_3N) to give 367 mg of compound **6a** (78%) as a white foam. TLC (6% MeOH in CH_2Cl_2 + 1% Et_3N), R_f 0.53.

^{1}H-NMR ($CDCl_3$, 400 MHz): δ 1.03 (t, 3H $^3J_{HH}$ = 7.13 Hz, H_3C), 1.56 (bs, 2H, $H_2N(2)$), 3.67 (s, 2H, $H_2CC(7)$); 3.72 (q, 2H, $^3J_{HH}$ = 7.24 Hz, $H_2CO(6)$), 3.76 & 3.79 (s, 12H, H_3CO(DMTr)); 5.40 (bs, 1H, HN(2)), 6.42 (s, 1H, CH(8)), 6.67-6.70 & 6.71-6.77 & 7.01-7.16 & 7.21-7.23 (m, 26H, HC(aromatic, DMTr) ppm.

^{13}C-NMR ($CDCl_3$, 101 MHz): δ 14.72 CH_3; 39.07 $CH_2C(7)$; 55.29 & 55.32 CH_3O(DMTr), 61.48 $CH_2O(6)$; 70.19 & 74.46 C(aromatic, DMTr), 99.44 C(5) / C(7); 112.65 & 112.70 C(aromatic, DMTr), 116.31 C(5) / C(7), 121.21 C(8), 126.14 & 126.76 & 127.36 & 127.39 & 129.17 & 130.01 & 130.35 & 131.46 & 135.94 & 139.11 & 143.70 C(aromatic, DMTr), 146.83 C (2), 155.91 C(4), 157.28 & 157.83 & 158.33 C(aromatic, DMTr), 162.15 C(6) ppm.

HR-ESI-MS (m/z): $[M+H]^+$ calculated 812.3806; found: 812.3785.

2.5. *O*^{6}-Ethyl preQ$_1$ (trifluoroacetate salt) (2a)

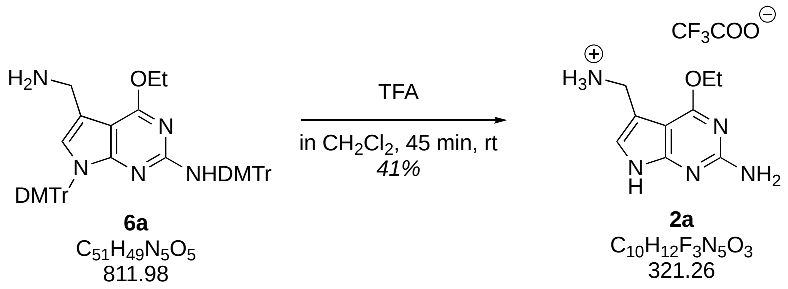

A solution of compound **6a** (105 mg, 0.13 mmol) in dichloromethane (1.0 ml) was treated with a mixture of trifluoroacetic acid (52 µl) and water (9 µl). The reaction was quenched by the addition of methanol (1.0 ml) after 15 min. Solvent was evaporated and the orange oil was coevaporated trice with methanol and dichloromethane respectively. The orange oil was further dried 2 h under high vacuum. The residue was then dissolved in dichloromethane and was left standing at ambient temperature overnight whereas the crude product precipitates as a white powder. The precipitate was collected by centrifugation and was purified by reversed phase chromatography (Lichoprep 0-20% ACN in water) to give 17 mg of compound **2a** as a white powder (41%). TLC (15% methanol in dichloromethane + 1% triethylamine), R_f 0.27.

^{1}H-NMR (DMSO-*d$_6$*, 700 MHz): δ 1.37 (t, 3H, $^3J_{HH}$ = 7.07 Hz, CH_3); 4.06 (q, 2H, $^3J_{HH}$ = 5.51 Hz, CH_2C(7)); 4.41 (q, 2H, $^3J_{HH}$ = 7.05 Hz, CH_2O(6)); 6.17 (bs, 2H, H_2N(2)), 6.92 (d, 1H, $^3J_{HH}$ = 2.22 Hz, HC(8)); 7.92 (bs s, 3H, NH_3^+); 11.17 (bs, 1H, HN(9)) ppm.

^{13}C-NMR (DMSO-*d$_6$*, 176 MHz): δ 14.44 CH_3, 35.16 CH_2CC(7); 61.10 CH_2O(6), 95.83 C(5) / C(7); 106.51 C(5) / C(7), 117.14 (***C***F_3COO^-, q, $^1J_{CF}$ = 299.84 Hz); 119.10 C (8); 154.82 C (2); 157.81 (CF_3***C***OO^-, q, $^2J_{CF}$ = 31.23 Hz); 159.76 C(4), 162.97 C(6) ppm.

ESI-MS: $[M+H]^+$ calculated: 208.1193, found: 208.1191; $[M+H-NH_3]^+$ calculated 191.0927; found: 191.0926.

2.6. NMR spectra of compounds 1a, 4a, 5a, 6a, and 2a

^{1}H-NMR (400 MHz, DMSO-d_6) of compound **1a**:

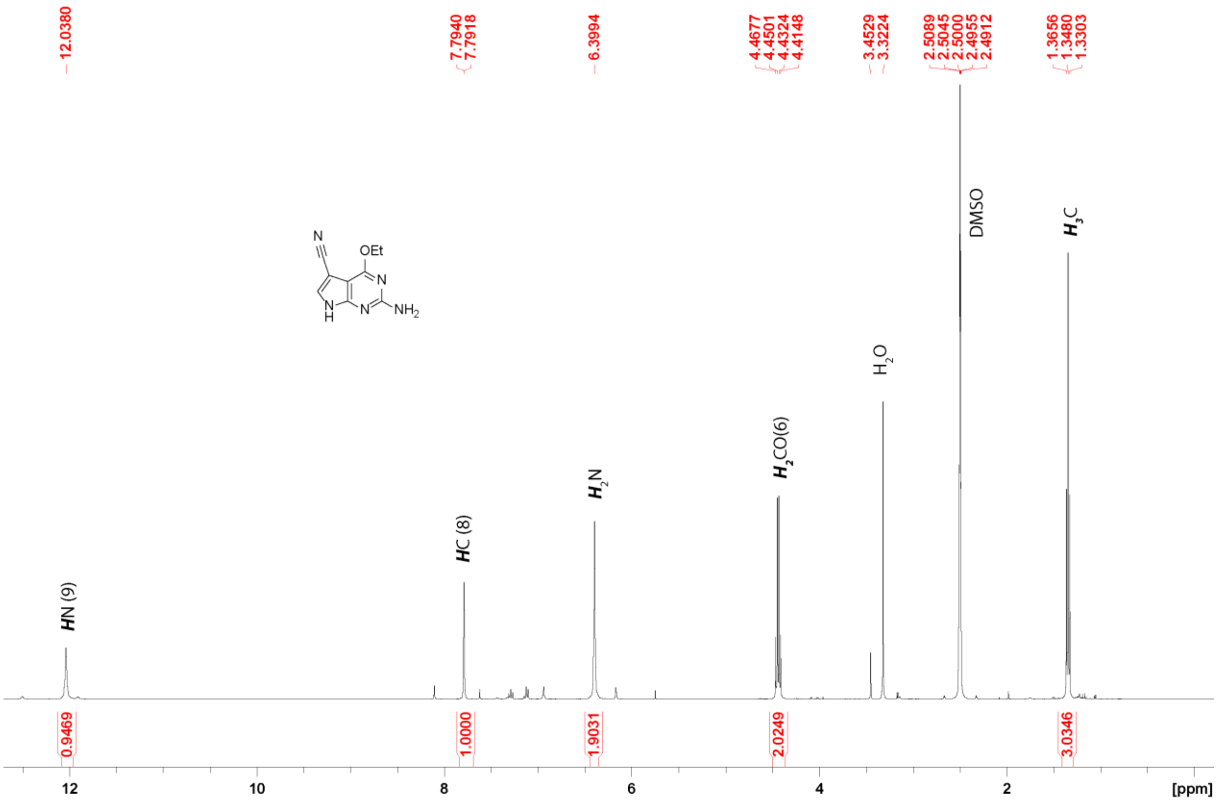

^{13}C-NMR (101 MHz, DMSO-d_6) of compound **1a**:

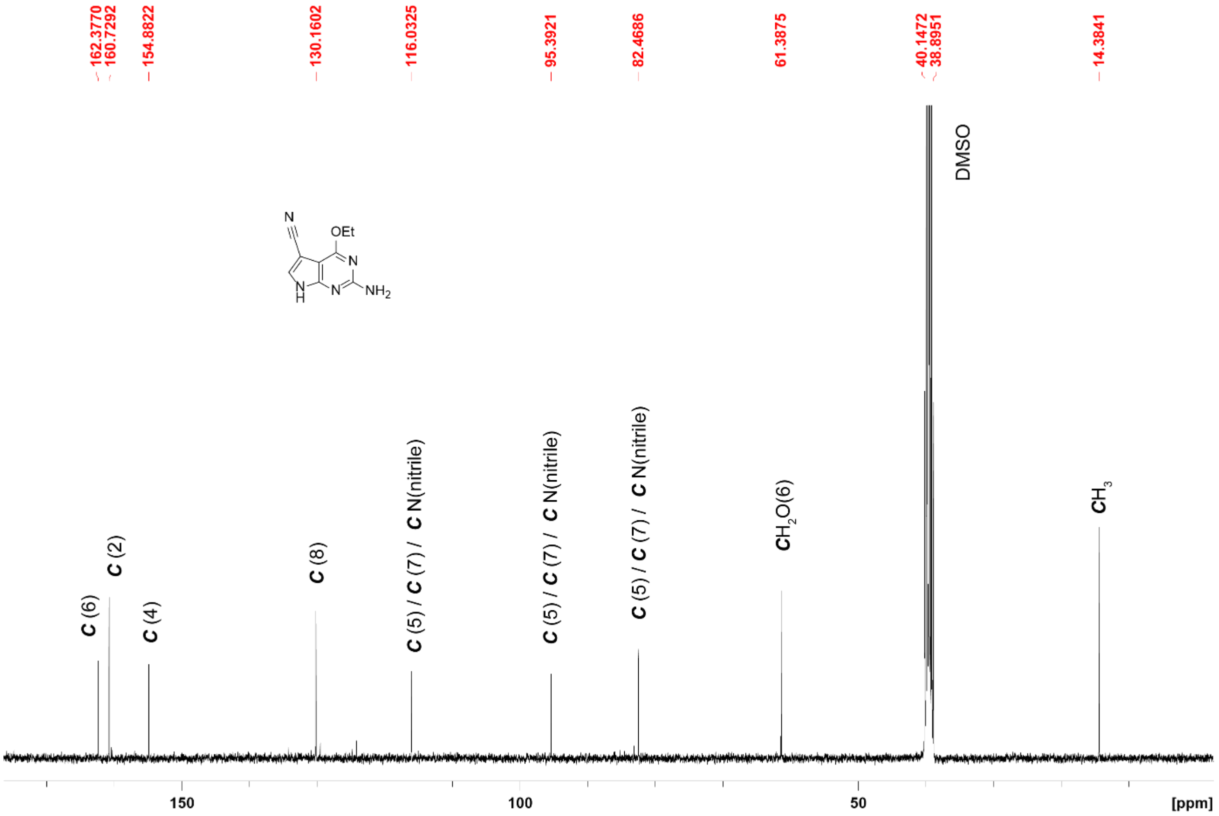

^1H-^{13}C HSQC NMR (400 MHz, DMSO-d_6) of compound **1a**:

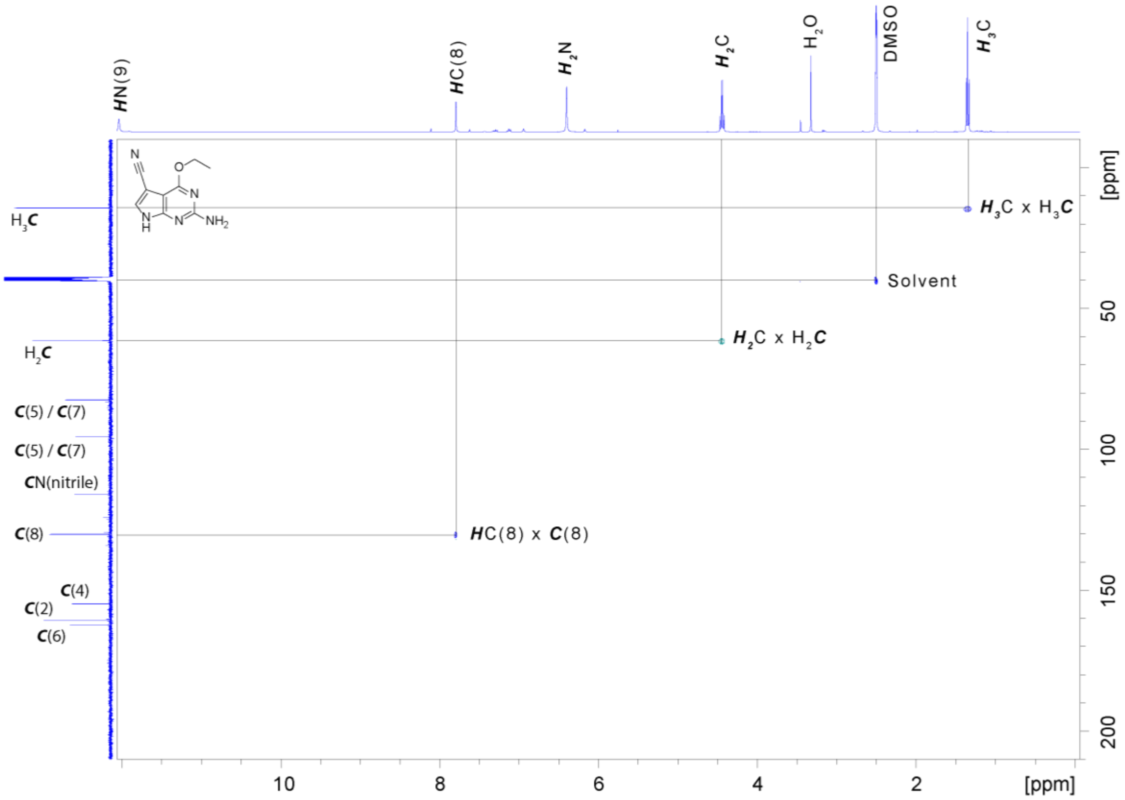

^1H-^{13}C HSQC NMR (400 MHz, DMSO-d_6) of compound **1a**:

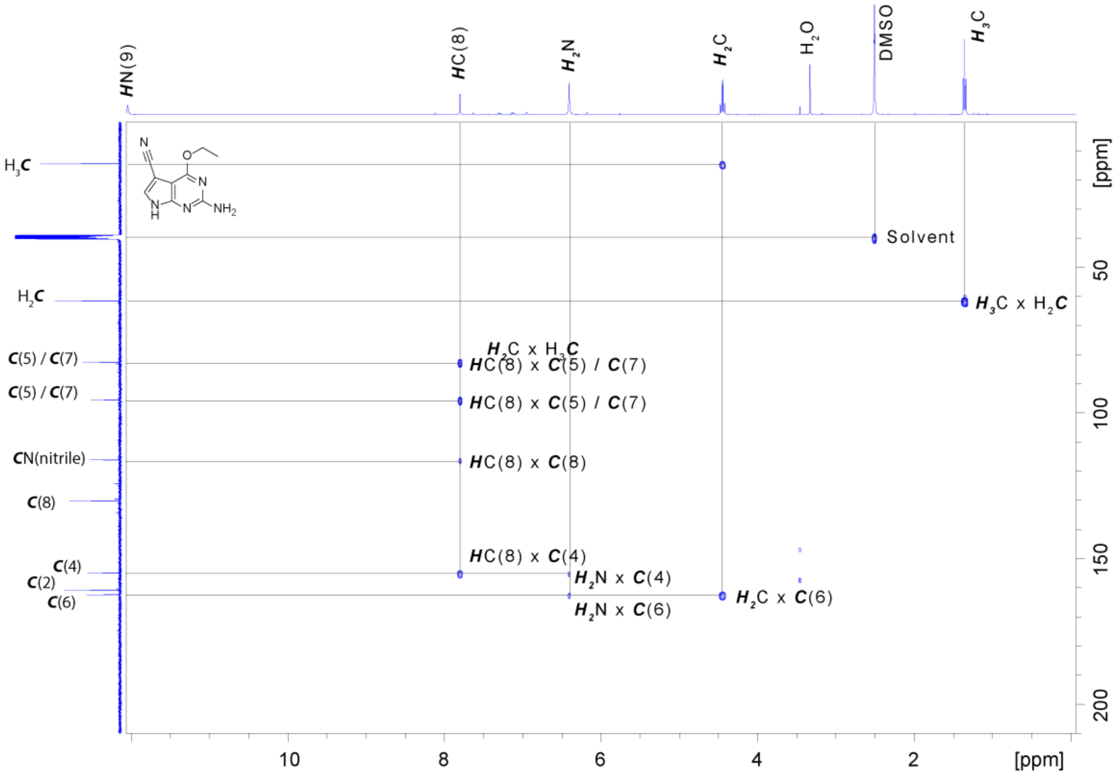

^{1}H-NMR (400 MHz, $CDCl_3$) of compound **4a**:

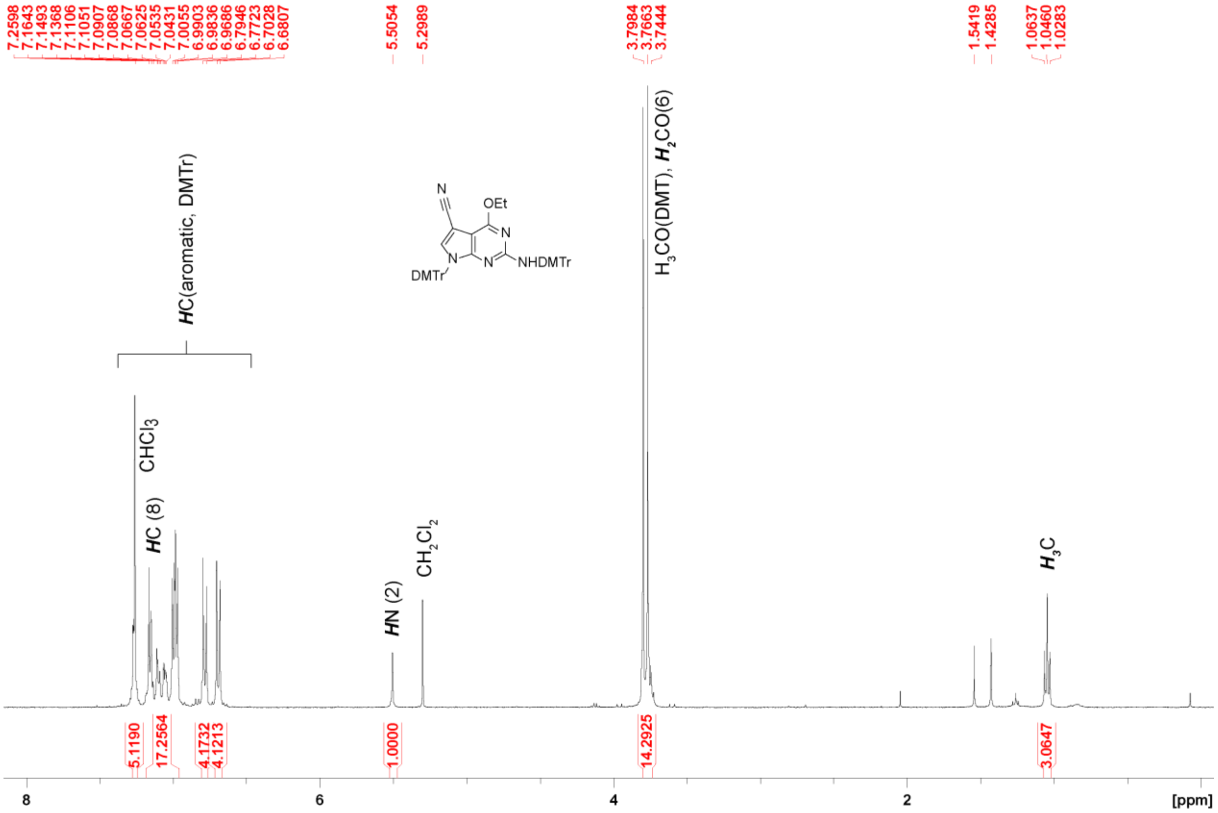

^{13}C-NMR (101 MHz, $CDCl_3$) of compound **4a**:

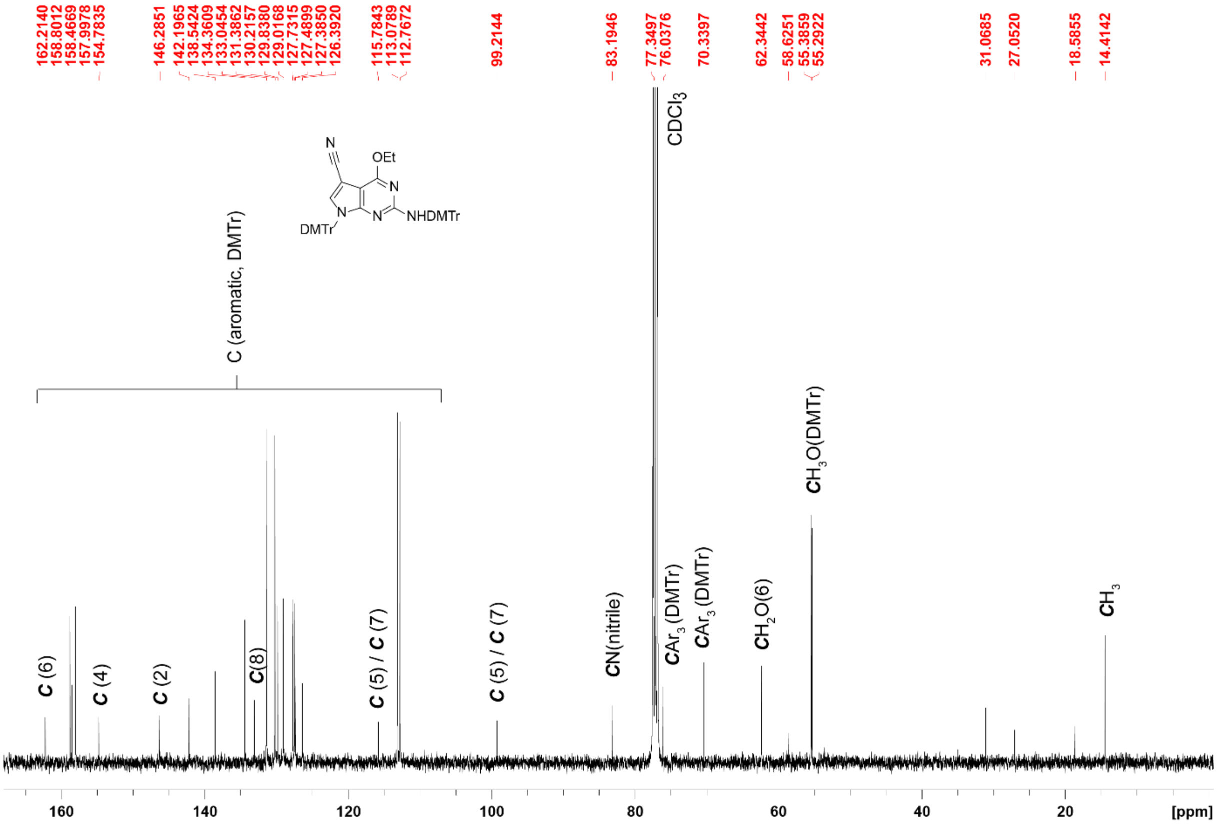

^{1}H-NMR (400 MHz, $CDCl_3$) of compound **5a**:

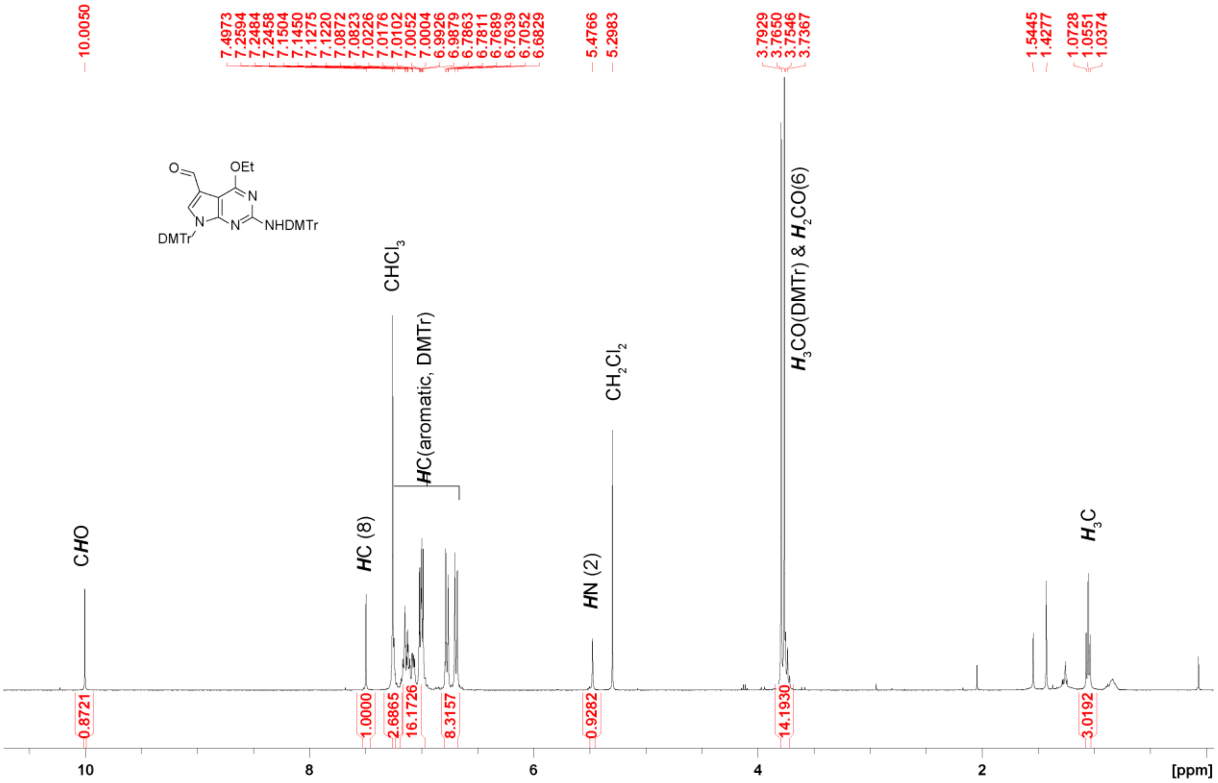

^{13}C-NMR (101 MHz, $CDCl_3$) of compound **5a**:

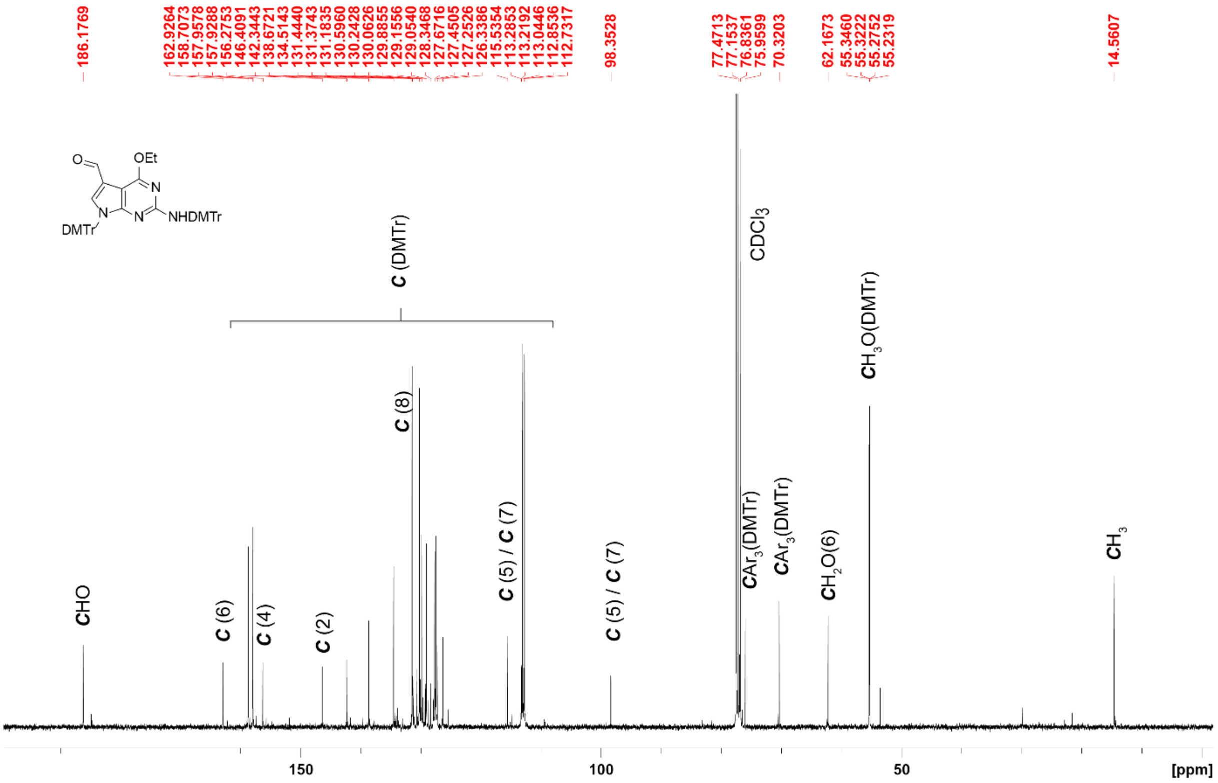

^{1}H-NMR (400 MHz, $CDCl_3$) of compound **6a**:

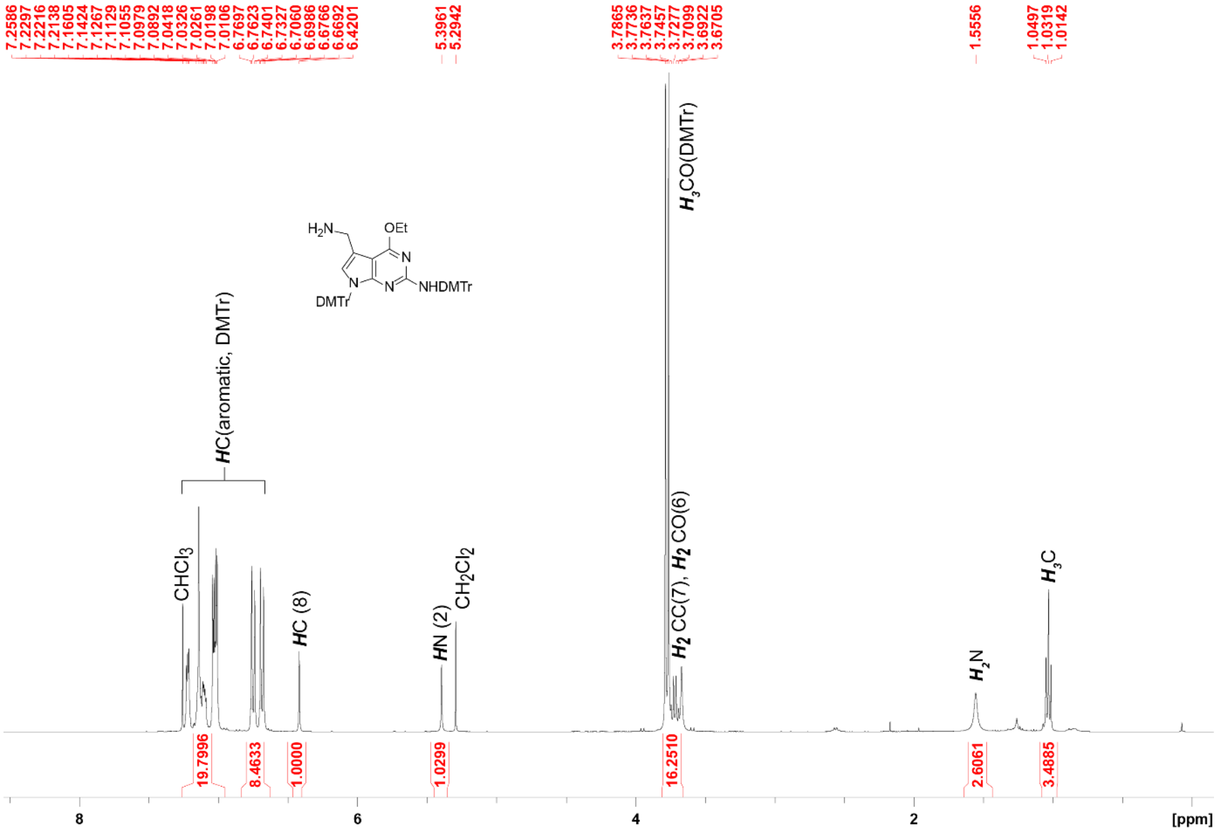

^{13}C-NMR (101 MHz, $CDCl_3$) of compound **6a**:

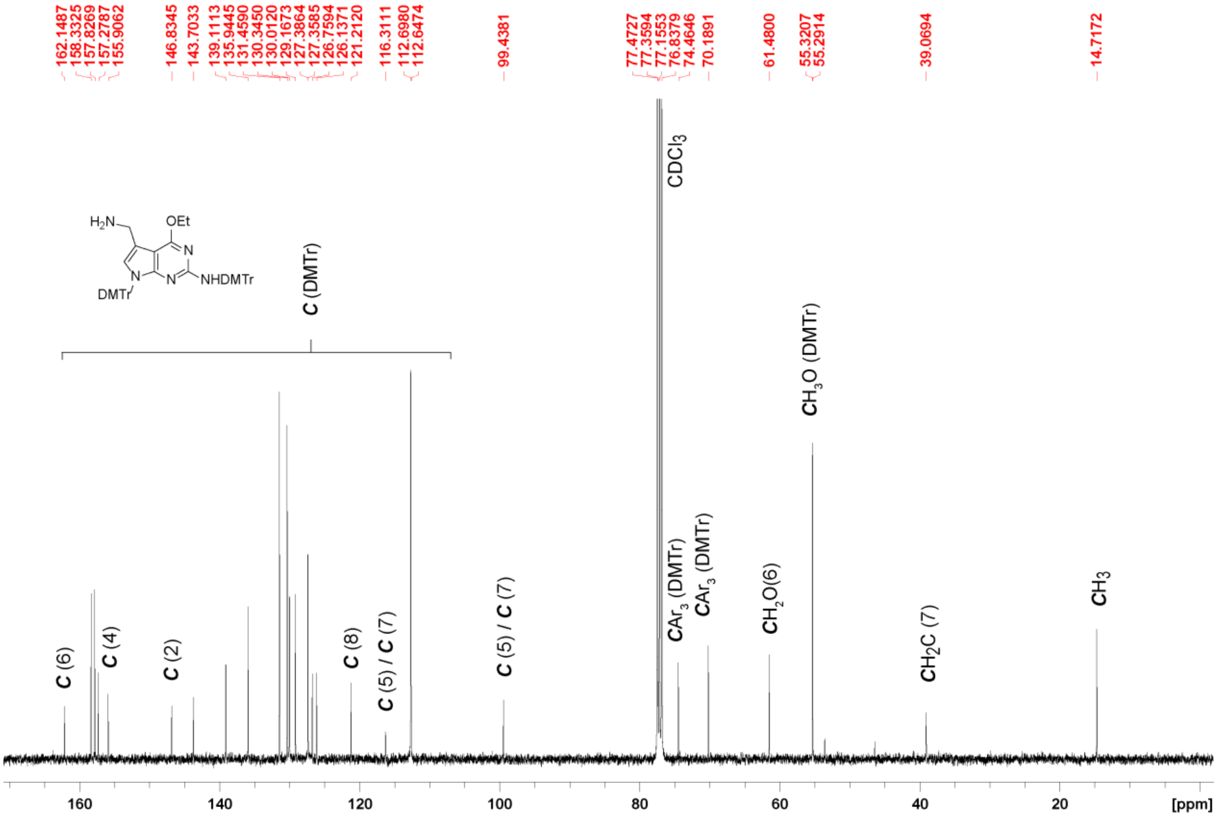

^{1}H-NMR (DMSO-d_6, 700 MHz) of compound **2a**:

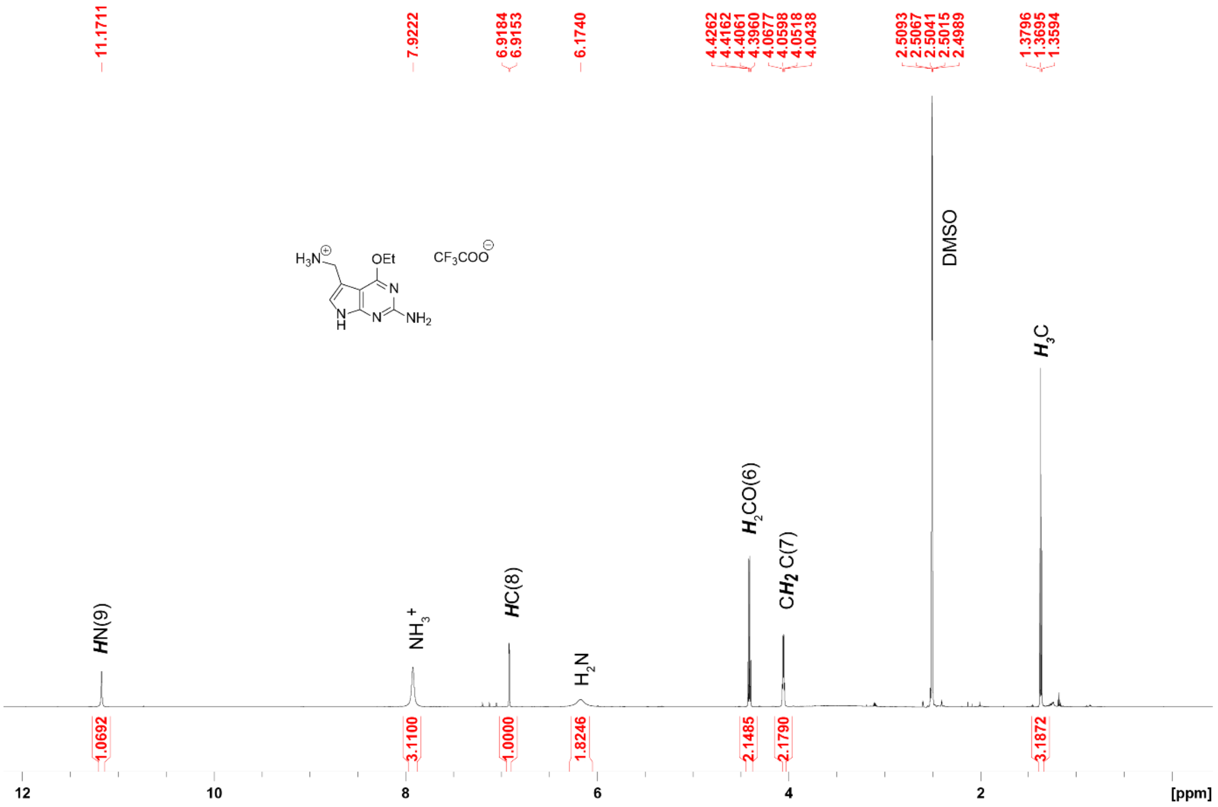

^{13}C-NMR (DMSO-d_6, 176 MHz) of compound **2a**.

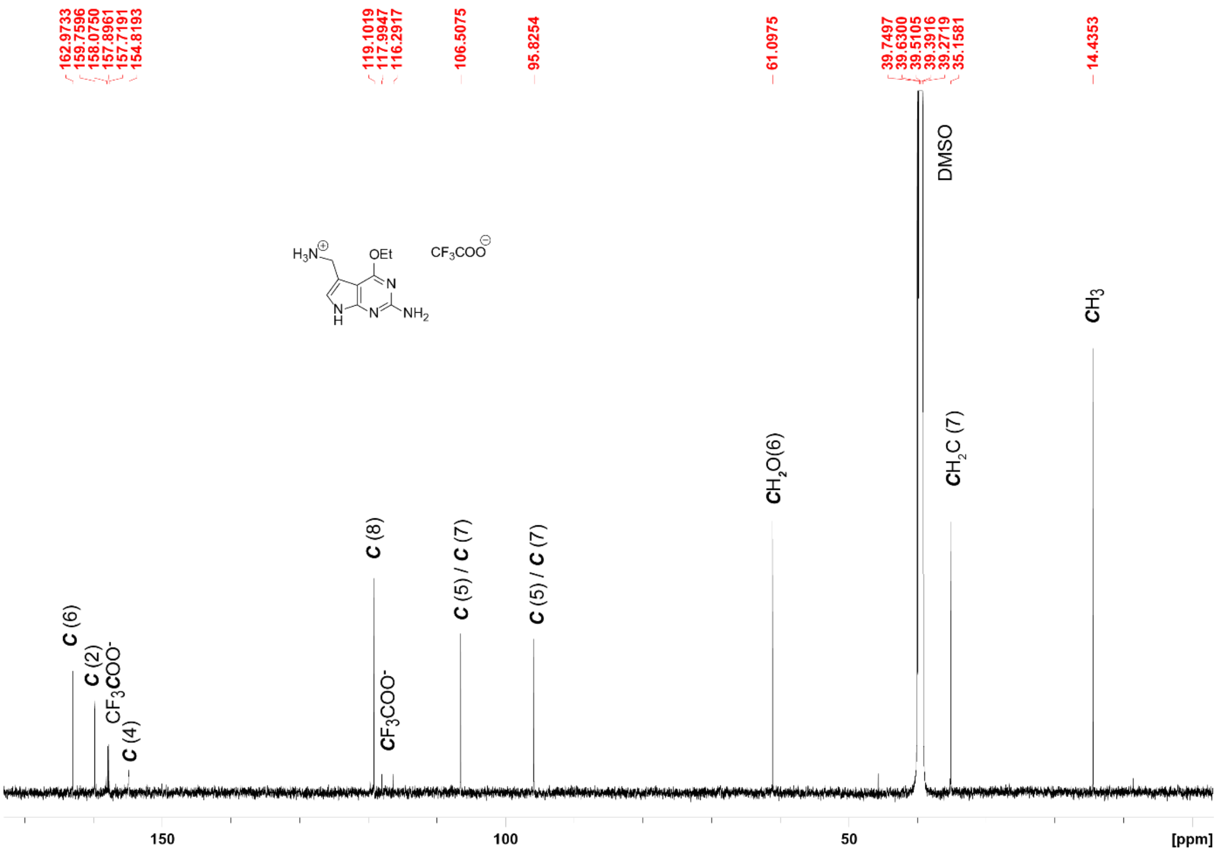

^{1}H-^{13}C HSQC NMR (400 MHz, DMSO-d_6) of compound **2a**:

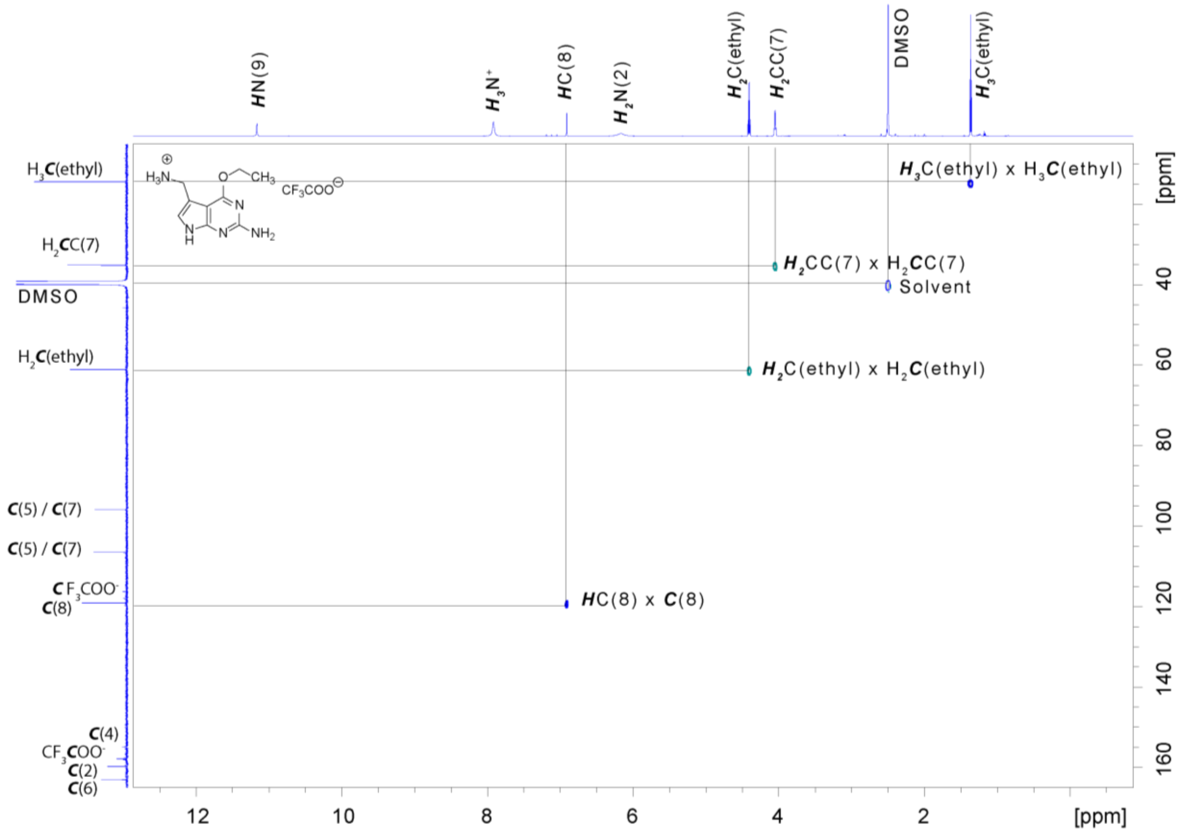

^{1}H-^{13}C HMBC NMR (400 MHz, DMSO-d_6) of compound **2a**:

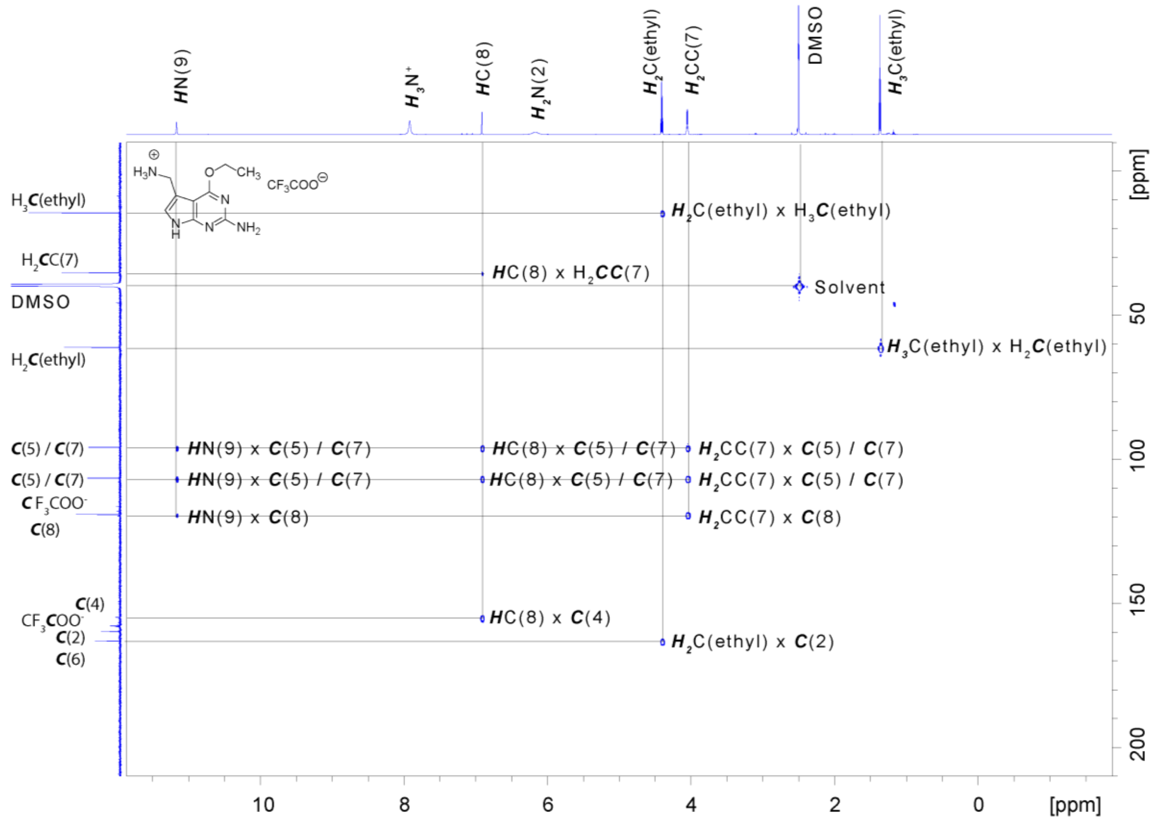

3. Synthesis of O^6-benzyl preQ$_1$ (trifluoroacetate salt) (2b)

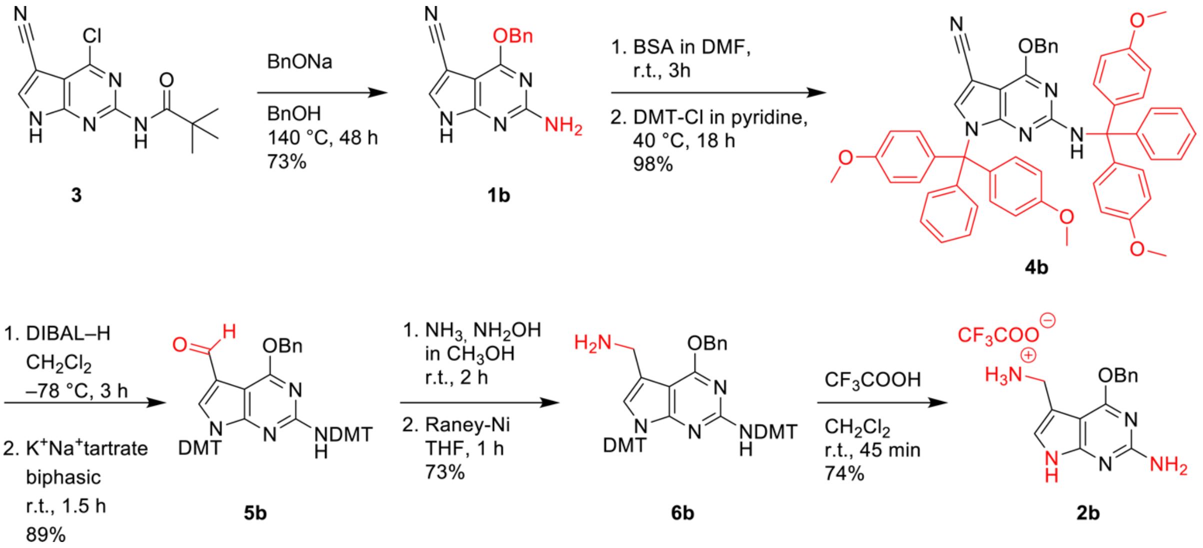

Supporting Scheme 3. Synthetic overview of the trifluoroacetate salt of bn^{6}preQ$_1$ **2b**.

3.1. *O*^{6}-Benzyl preQ$_0$ (1b)

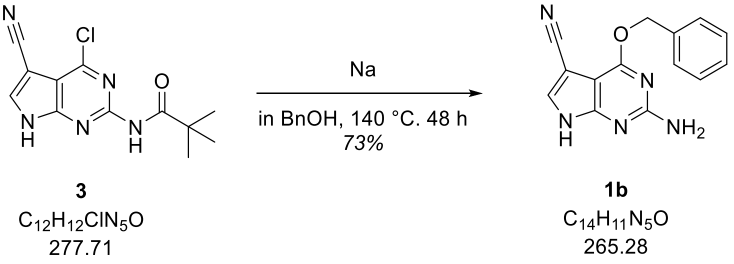

Sodium (300 mg, 1.14 mmol) was dissolved in 5.5 mL benzyl alcohol at ambient temperature. To this solution compound **3** (300 mg, 1.08 mmol) was added and the mixture was heated to 140 °C for 48 h. Afterwards, the benzyl alcohol was distilled off and the remaining solid was suspended in methanol and dry-loaded onto silica gel. Flash column chromatography (1–5% methanol in dichloromethane) provided 210 mg of compound **1b** (73%) as a white solid. TLC: 8% methanol in dichloromethane, R_f 0.5.

^{1}H-NMR (DMSO-d$_6$, 300 MHz): δ 12.1 (bs, 1H, HN(9)), 7.83 (d, 1H, J_{HH} = 1.9 Hz, HC(8)), 7.56-7.47 (m, 2H, HC(aromatic, benzyl)), 7.44-7.29 (m, 3H, HC(aromatic, benzyl)), 6.50 (bs, 2H, H$_2$N(2)), 5.52 (s, 1H, H$_2$C(benzyl)) ppm.

^{13}C-NMR (DMSO-d$_6$, 75 MHz): δ 162.1 & 160.7 C(2) & C(6), 155.1 C(4), 136.7 C(aromatic, benzyl), 130.7 C(8), 128.4 C(aromatic, benzyl), 127.7 C(aromatic, benzyl), 127.4 C(aromatic, benzyl), 116.0 & 95.5 C(7) & C(nitrile), 82.4 C(5), 66.5 CH$_2$(benzyl) ppm.

ESI-MS: [M+H]$^+$ found 266.1032; [M+H]$^+$ calculated 266.1036.

3.2. N^2,9-Bis-(4,4'-dimethoxytrityl)-O^6-benzyl preQ$_0$ (4b)

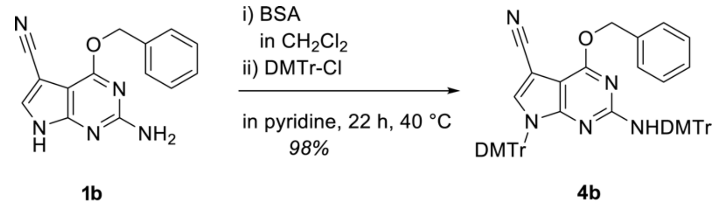

1b
$C_{14}H_{11}N_5O$
265.28

4b
$C_{56}H_{47}N_5O_5$
870.02

Compound **1b** (40.0 mg, 151 µmol) was suspended in dichloromethane (500 µL). *N,O*-bis(trimethylsilyl)-acetamide (80 µL, 320 µmol) was slowly added. After a few minutes a homogenous solution was obtained and the mixture was stirred for another one and a half hour at room temperature. The volatiles were removed in vacuo and the residue was dissolved in pyridine (600 µL). 4,4'-dimethoxytrityl chloride (118 mg, 247 µmol) was added and the solution was stirred for 20 h at 40 °C. Aqueous sodium bicarbonate solution (5%, 10 mL) was added and the mixture was extracted three times with ethyl acetate. After drying over magnesium sulfate the crude mixture was purified by flash column chromatography on silica gel (20% ethyl acetate in cyclohexane) to give 129 mg of compound **4b** (98%) as a white foam. TLC: 30% ethyl acetate in cyclohexane, R_f 0.60.

^{1}H-NMR ($CDCl_3$, 400 MHz): δ 7.33-7.25 & 7.23-7.13 & 7.11-7.06 & 7.06-6.99 & 6.93-6.79 & 6.73-6.69 (m, 32H, HC(aromatic, DMTr) & HC(aromatic, benzyl) & HC(8)), 5.57(s, 1H, HN(2)), 4.76 (s, 2H, H_2C(benzyl)), 3.81 & 3.77 (s, 12H, H_3CO(DMTr)) ppm.

^{13}C-NMR ($CDCl_3$, 101 MHz): δ 161.8 C(6), 158.8 & 158.3 & 158.0 & 154.9 C(aromatic, DMTr & benzyl), 146.2 C(2), 142.1 C(aromatic, DMTr & benzyl), 138.4 C(4), 136.7 & 134.3 C(aromatic, DMTr & benzyl), 133.2 C(8), 131.4 & 130.2 & 129.8 & 129.3 & 129.0 & 128.4 & 127.8 & 127.6 & 127.4 & 127.2 & 126.5 C(aromatic, DMTr & benzyl), 115.7 C(5) or C(7) or CN(nitrile), 113.1 & 112.8 C(aromatic, DMTr & benzyl), 99.3 & 83.3 C(5) or C(7) or CN(nitrile), 76.1 & 70.4 CAr_3(DMTr), 67.4 CH_2(benzyl), 55.4 & 55.3 CH_3O(DMTr) ppm.

ESI-MS: $[M+H]^+$ found 870.3630; $[M+H]^+$ calculated 870.3650.

3.3. 7-Formyl-N^2,9-bis(4,4'-dimethoxytrityl) O^6-benzyl-7-deazaguanine (5b)

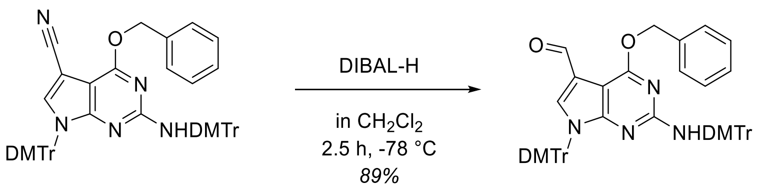

4b
$C_{56}H_{47}N_5O_5$
870.02

5b
$C_{56}H_{48}N_4O_6$
873.02

Compound **4b** (100 mg, 115 µmol) was dissolved in dichloromethane (750 µL) and cooled to –78 °C. After addition of diisobutylaluminium hydride (150 µL, 1.0 M solution in dichloromethane) the mixture was stirred for two hours at this temperature. The reaction was quenched upon the addition of ethyl acetate (2 mL) and allowed to warm to room temperature. Half-saturated aqueous sodium potassium tartrate solution (2 mL) was added and vigorously stirred for two hours. The aqueous and organic

phase were separated and the aqueous phase was extracted twice with ethyl acetate. The combined organic phases were dried over magnesium sulfate and purified by flash column chromatography on silica gel (20% ethyl acetate in cyclohexane) to give 90 mg of compound **5b** (89%) as a white foam. TLC: 30% ethyl acetate in cyclohexane, R_f 0.53

^{1}H-NMR ($CDCl_3$, 400 MHz): δ 10.00 (s, 1H, CHO), 7.52 (s, 1H, HC(8)), 7.34-7.24 & 7.22-7.13 & 7.12-7.08 & 7.07-7.00 & 7.82-6.77 & 6.73-6.68 (m, 31H, HC(aromatic)), 5.53 (s, 1H, HN(2)), 4.72 (s, 2H, H_2C(benzyl)), 3.80 (s, 6H, H_3CO(DMTr)), 3.76 (s, 6H, H_3CO(DMTr)) ppm.

^{13}C-NMR ($CDCl_3$, 101 MHz): δ 186.0 CHO, 162.6 C(6), 158.8 &158.0 & 157.8 C(aromatic), 156.5 C(4), 146.4 C(2), 142.3 & 138.6 & 136.8 & 134.5 & 131.5 C(aromatic), 130.9 C(8), 130.3 & 130.0 & 129.1 & 128.5 & 128.0 & 127.9 & 127.8 & 127.7 & 127.6 & 127.5 & 127.3 & 126.4 C(aromatic), 115.5 C(7), 113.3 & 113.1 & 112.8 C(aromatic), 98.3 C(5), 76.1 CAr_3(DMTr), 70.4 CAr_3(DMTr), 67.8 CH_2(benzyl), 55.4 & 55.3 CH_3O(DMTr) ppm.

ESI-MS: $[M+H]^+$ found 873.3624; $[M+H]^+$ calculated 873.3647.

3.4. 7-Aminomethyl-*N*^{2},9-bis(4,4'-dimethoxytrityl)-*O*^{6}-benzyl-7-deazaguanine (6b)

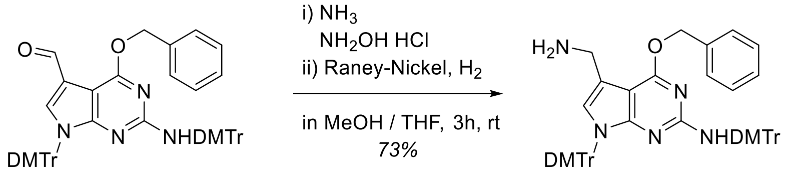

5b $C_{56}H_{48}N_4O_6$ 873.02 — **6b** $C_{56}H_{51}N_5O_5$ 874.05

Compound **5b** (300 mg, 345 µmol) was suspended in 1.5 mL 7 M ammonia in methanol, hydroxylamine hydrochloride (29 mg, 410 µmol) and tetrahydrofuran (0.5 mL) were added and the mixture was stirred for one and a half hour at room temperature. Damp Raney-Nickel (approximately 100 mg) was added and hydrogen was bubbled through the solution for half an hour under vigorous stirring. Bubbling was stopped and the reaction was continued for one hour, after which the mixture was filtered over a Celite pad and the filtrate was concentrated in vacuo. Purification via silica gel column chromatography (1-3% methanol in dichloromethane) afforded 218 mg of compound **6b** (73%) as a white foam. TLC: 6% methanol in dichloromethane, R_f0.46.

^{1}H-NMR ($CDCl_3$, 400 MHz): δ 7.31-2.1 & 7.19-7.09 & 7.08 – 6.97 & 6.79-6.72 & 6.71-6.64 (31H, m, HC(aromatic)), 6.45 (s, 1H, HC(8)), 5.43 (s, 1H, HN(2)), 4.69 (s, 2H, H_2C(benzyl)), 3.79 & 3.76 (s, 12H, H_3CO(DMTr), 3.68 (s, 1H, H_2CC(7)), 2.62 (bs, 2H, H_2N).

^{13}C-NMR ($CDCl_3$, 101 MHz): δ 161.86 C(6), 158.36 & 157.87 & 157.14 C(aromatic, DMTr, benzyl), 156.05 C(4), 146.78 C(2), 143.63 & 138.98 & 137.33 & 135.86 & 131.45 & 130.33 & 130.00 & 129.15 & 128.50 & 127.73 & 127.69 & 127.44 & 127.42 & 126.81 & 126.23 C(aromatic, DMTr, benzyl), 121.57 C(8), 115.93 C(5) / C(7), 112.72 C(aromatic, DMTr, benzyl), 99.34 C(5) / C(7), 74.56 & 70.23 (CAr_3(DMTr)), 67.25 H_2C(benzyl), 55.32 & 55.27 CH_3O(DMTr), 38.95 H_2CC(7) ppm.

ESI-MS: $[M+H]^+$ found 874.3942; $[M+H]^+$ calculated 874.3963.

3.5. O^6-Benzyl preQ$_1$ (trifluoroacetate salt) (2b)

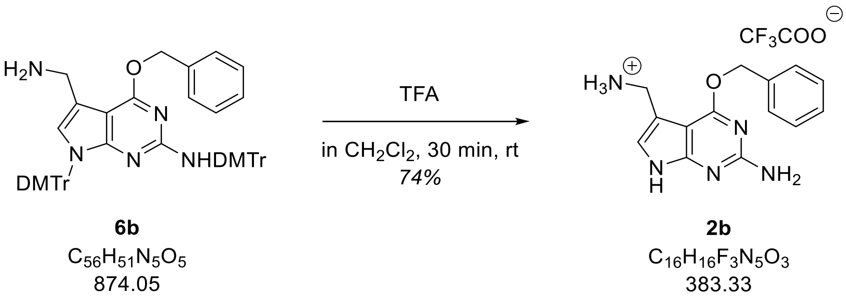

To a solution of compound **6b** (197 mg, 225 µmol) in dichloromethane (750 µL) were added trifluoroacetic acid (90 µL, 2.27 mmol) and water (15 µL). After 30 minutes stirring at room temperature the volatiles were removed in vacuo and the residual oil was coevaporated with methanol and dichloromethane. After short drying on high vacuum the crude product was made basic by the addition of triethylamine and purified by flash column chromatography on silica gel (5–15% methanol in dichloromethane). The pure product was dissolved in a small amount of methanol and trifluoroacetic acid (20 µL) was added. After evaporation and trituration with dichloromethane 64 mg of compound **2b** (74%) as a beige solid were obtained. TLC: 15% methanol in dichloromethane 1% triethylamine, R_f 0.35

^{1}H-NMR (DMSO-*d6*, 400 MHz): δ 11.34 (bs, 1H, HN(9)), 7.98 (bs, 3H, H_3N^+), 7.54-7.50 (m, 2H, H(arom, benzyl), 7.44-7.32 (m, 3H, H(arom, benzyl), 6.95 (d, 1H, J_{HH} = 2.2 Hz, HC(8)), 5.48 (s, 2H, H_2C(benzyl)), 4.06 (q, 2H, H_2C(C7)).

^{13}C-NMR (DMSO-*d6*, 101 MHz):: δ 163.4 C(6), 158.5 (q, J_{CF} = 34 Hz, CF_3**C**OO^-), 158.3 C(2), 151.8 C(4), 136.6 & 128.5 & 128.9 & 127.9 C(arom, benzyl), 119.7 C(8), 116.5 (q, J_{CF} = 296 Hz, **C**F_3COO^-), 107.3 & 96.1 C(5) & C(7), 67.1 H_2C(benzyl), 34.9 H_2**C**C(7).

ESI-MS: $[M+H]^+$ found 270.1343; $[M+H]^+$ calculated 270.1349; $[M+H-NH_3]^+$ found 253.1079; $[M+H-NH_3]^+$ calculated 253.1084.

3.6. NMR spectra of compounds **1b**, **4b**, **5b**, **6b**, and **2b**

^{1}H-NMR (300 MHz, DMSO-d_6) of compound **1b**:

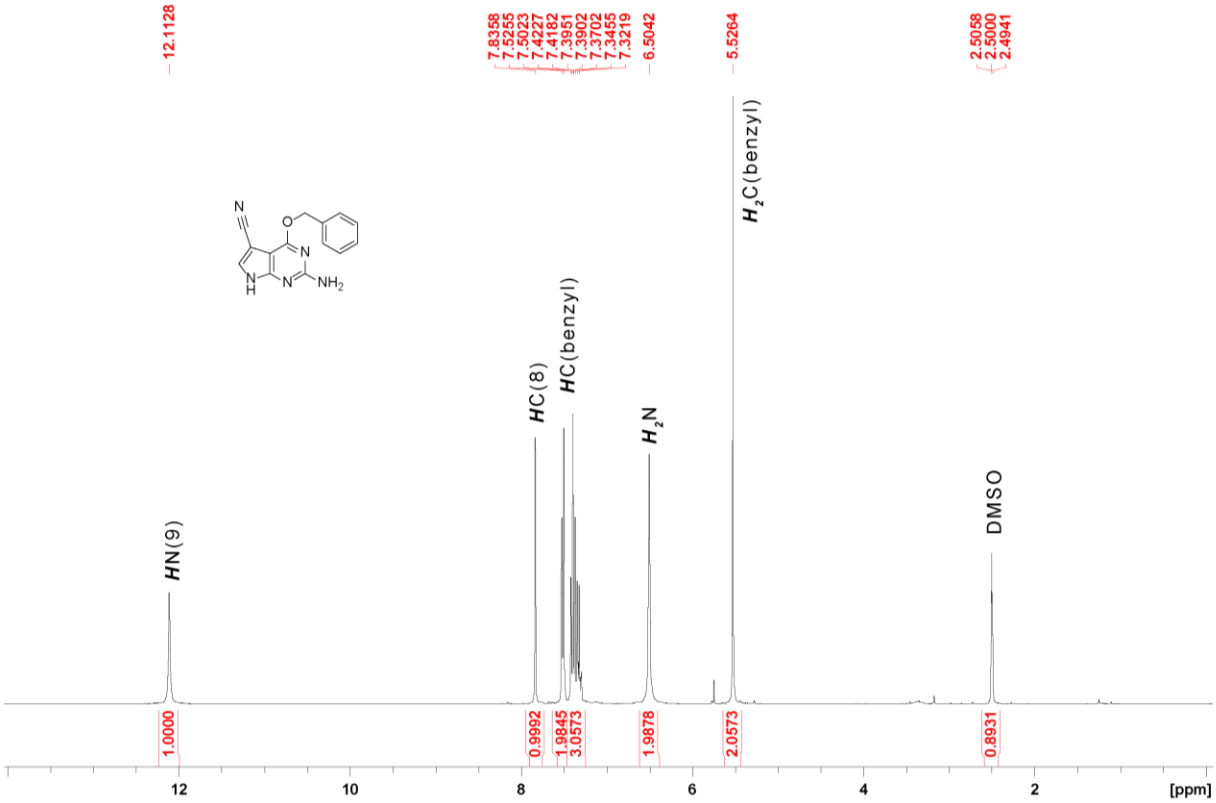

^{13}C-NMR (75 MHz, DMSO-d_6) of compound **1b**:

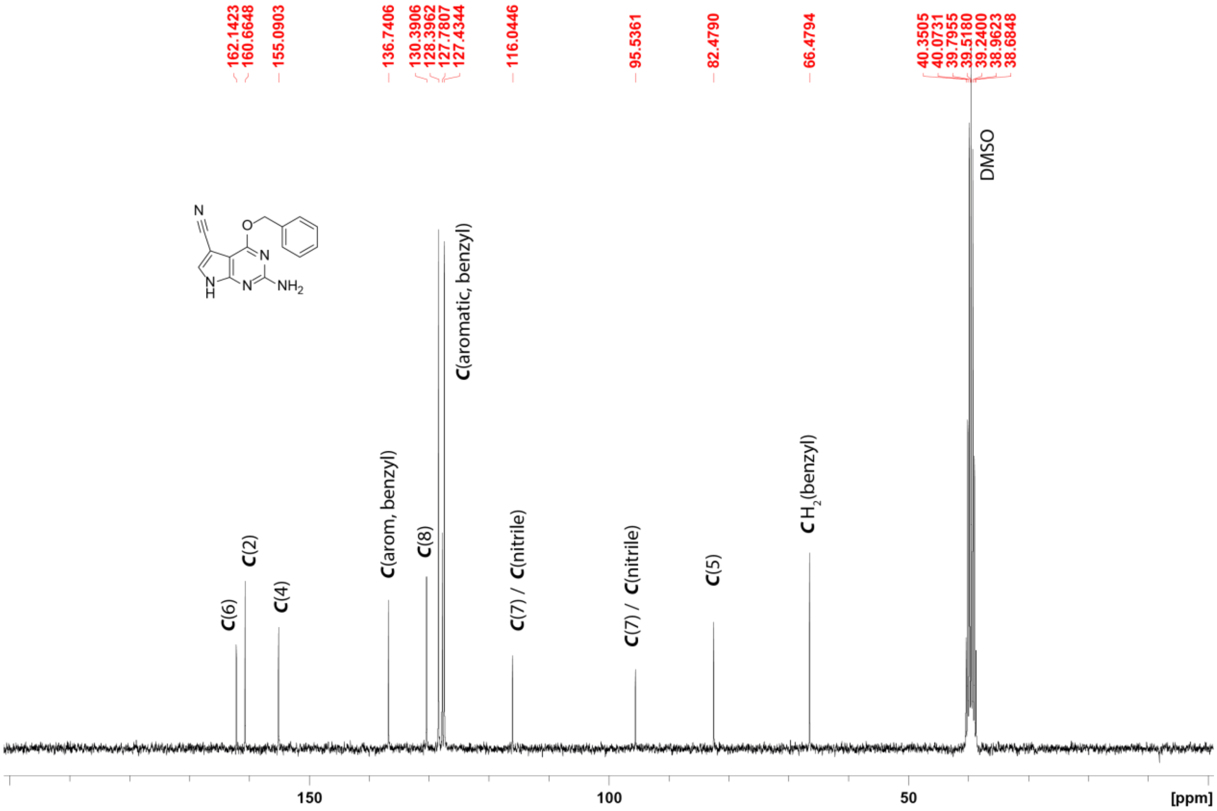

^{1}H-^{13}C HSQC NMR (300 MHz, DMSO-d_6) of compound **1b**:

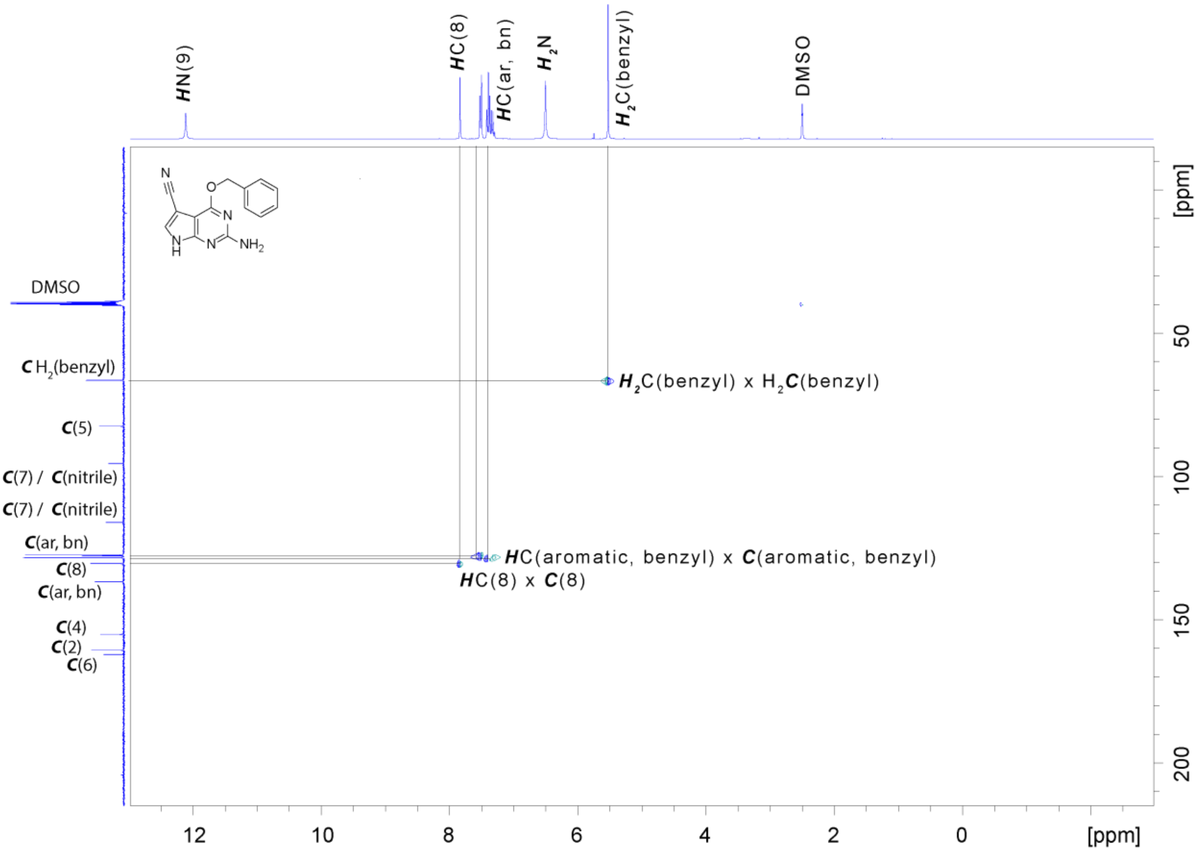

^{1}H -^{13}C HMBC NMR (300 MHz, DMSO-d_6) of compound **1b**:

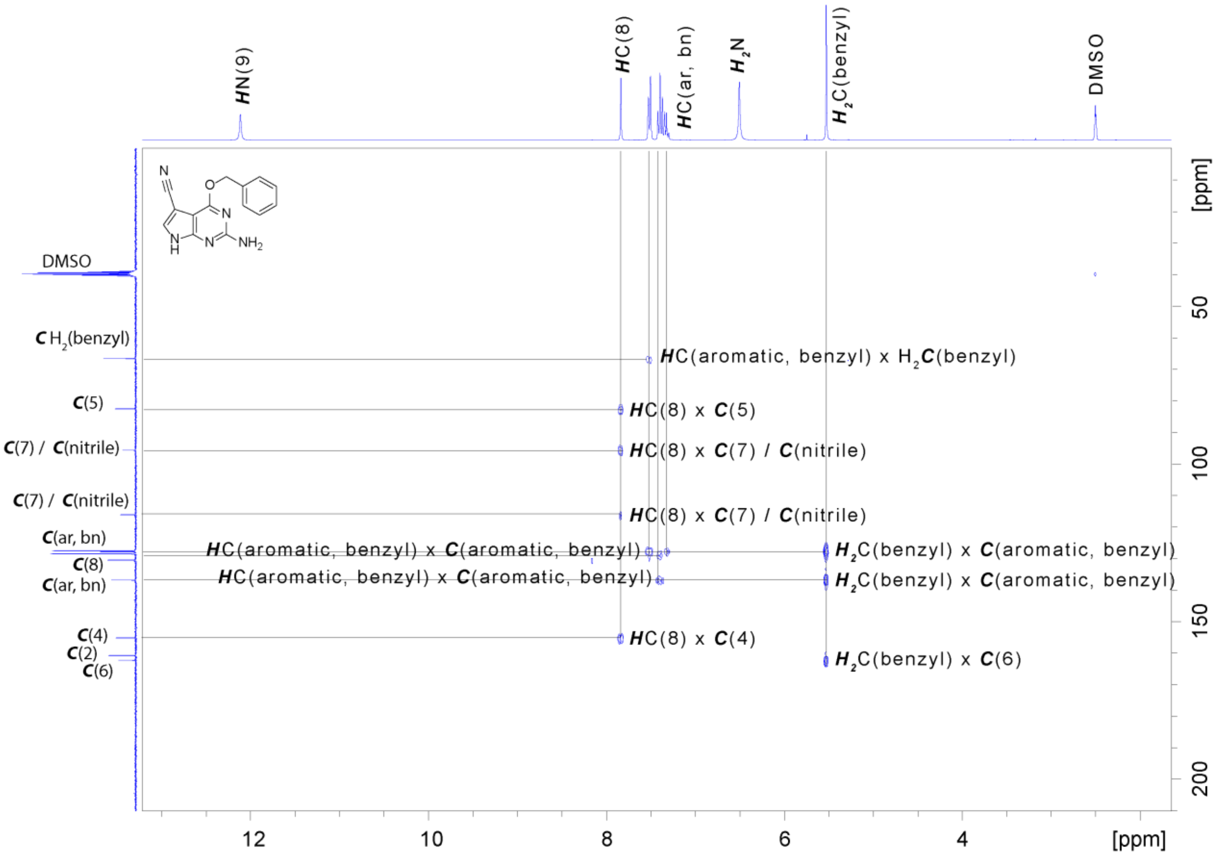

^{1}H-NMR (400 MHz, $CDCl_3$) of compound **4b**:

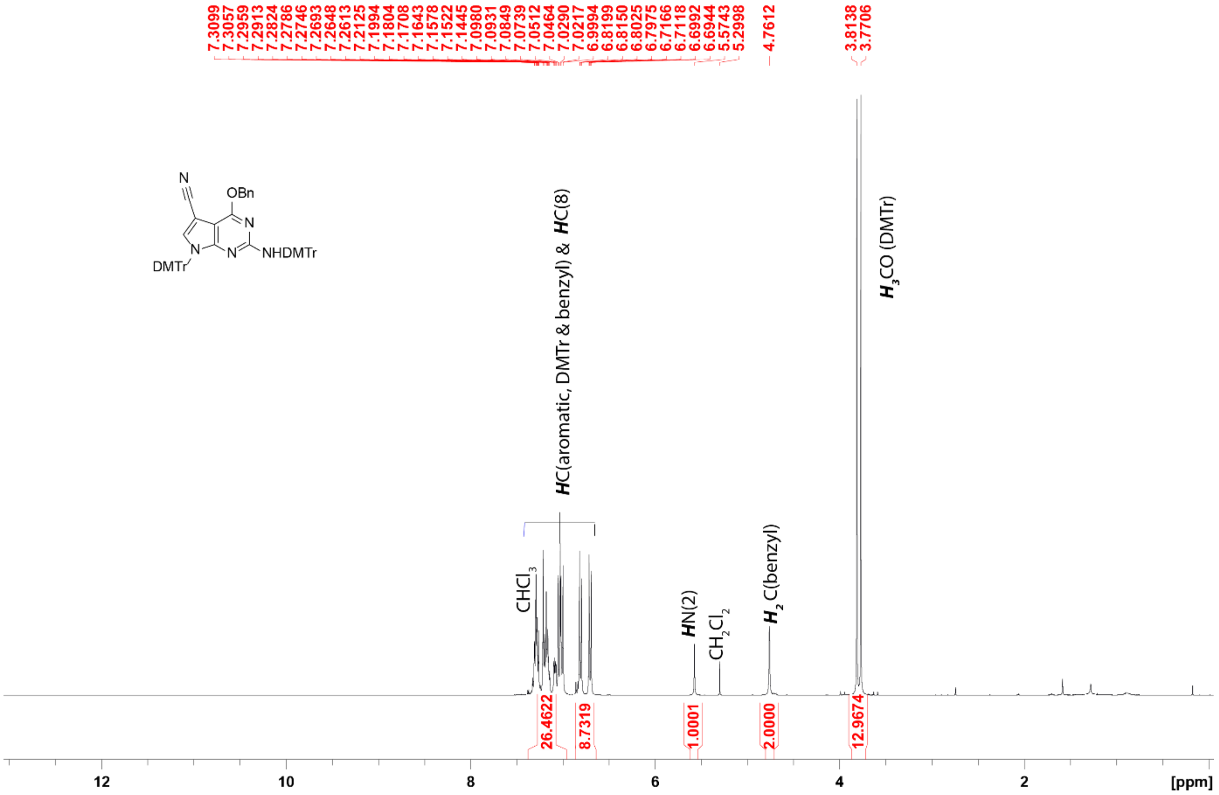

^{13}C-NMR (101 MHz, $CDCl_3$) of compound **4b**:

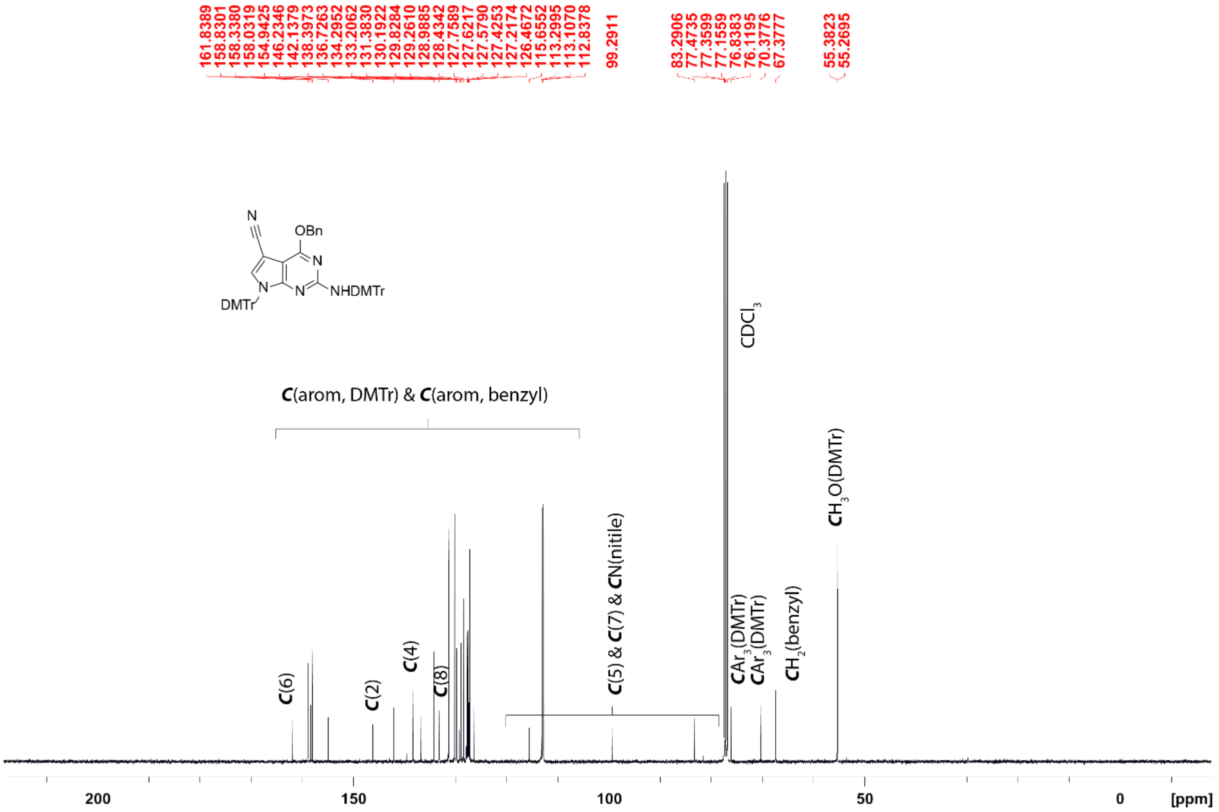

^{1}H-NMR (400 MHz, $CDCl_3$) of compound **5b**:

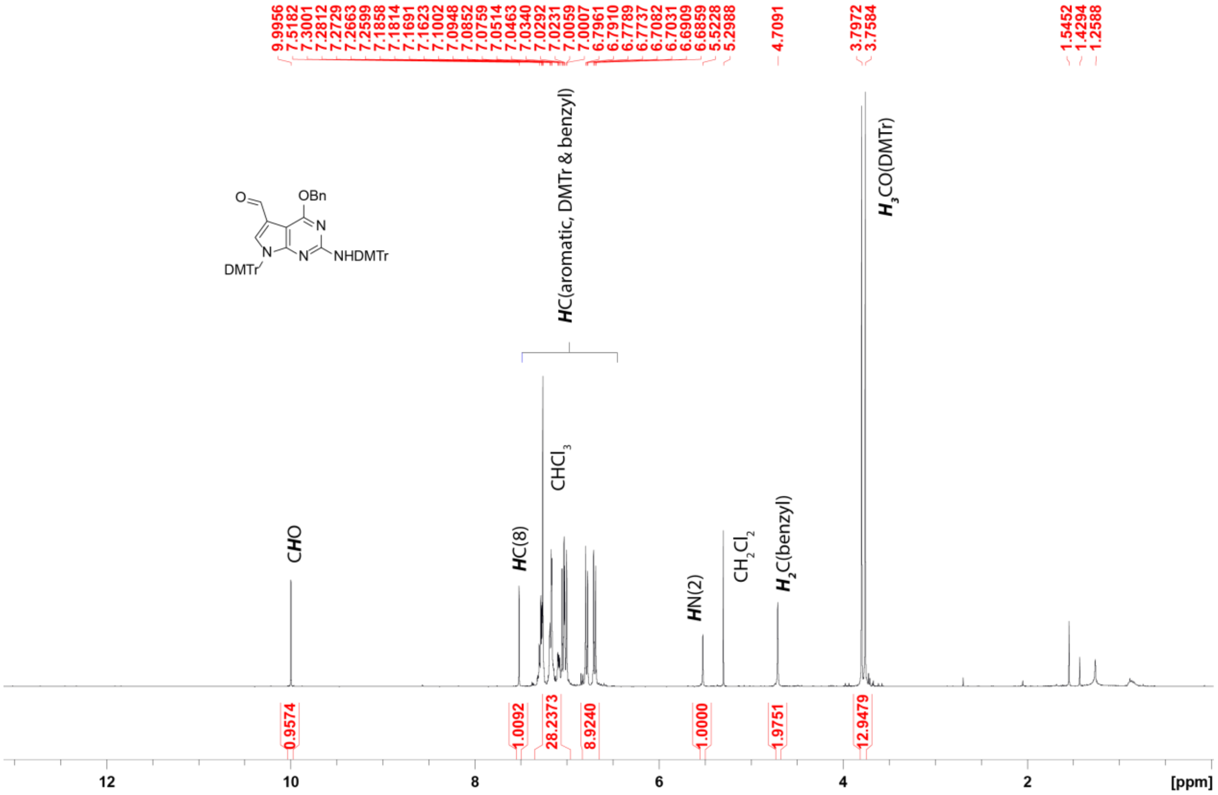

^{13}C-NMR (101 MHz, $CDCl_3$) of compound **5b**:

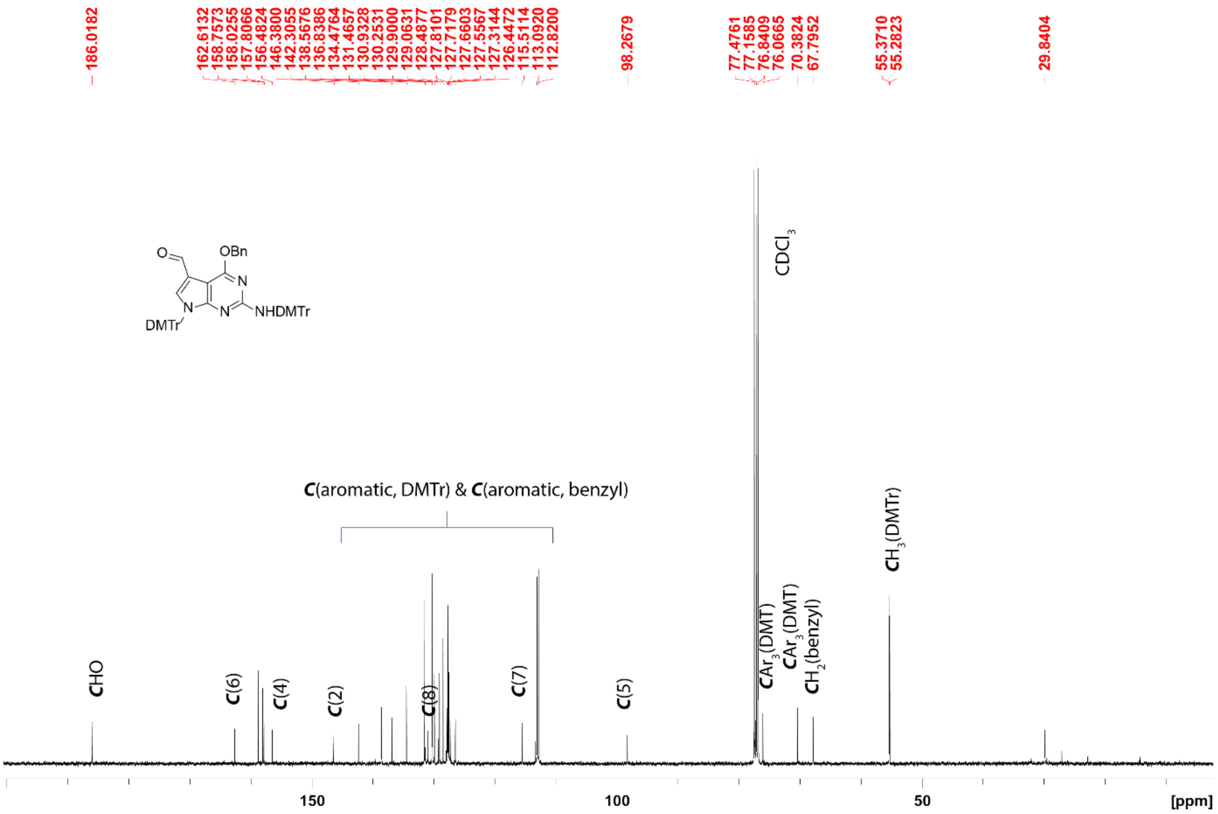

^{1}H-NMR (400 MHz, $CDCl_3$) of compound **6b**:

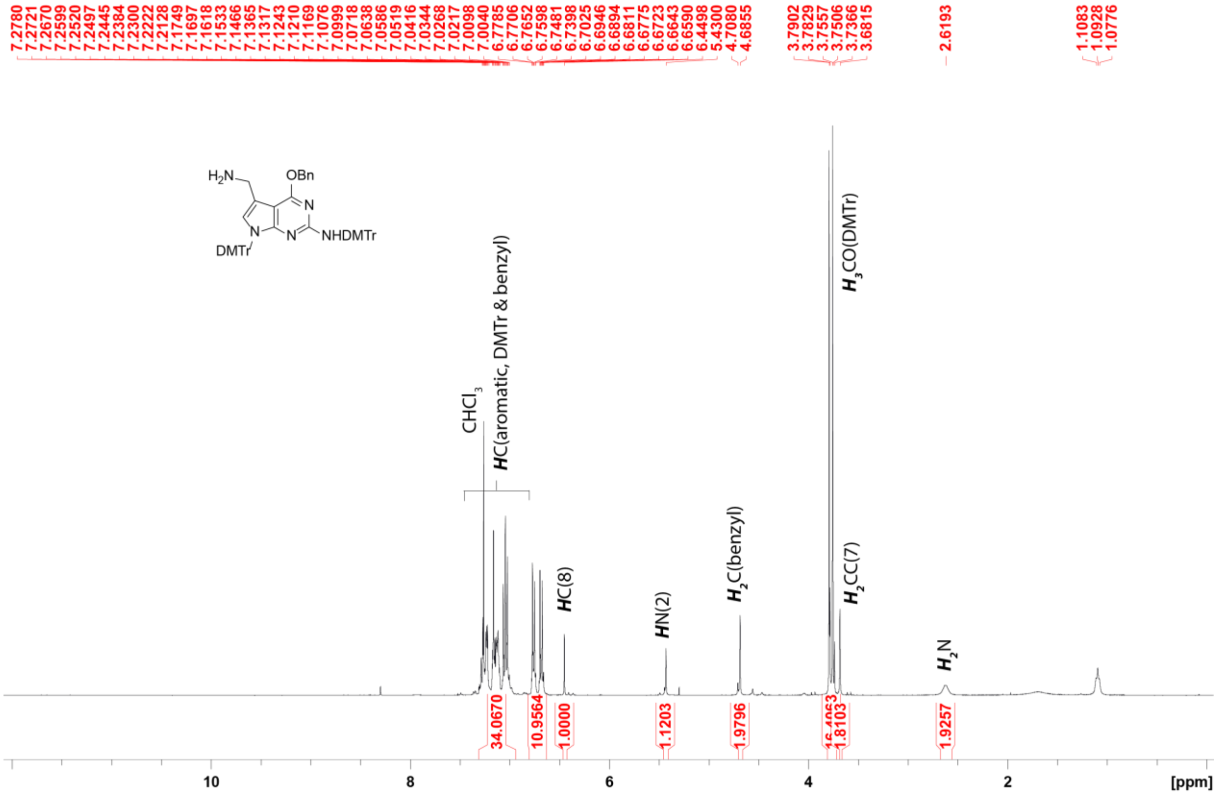

^{13}C-NMR (101 MHz, $CDCl_3$) of compound **6b**:

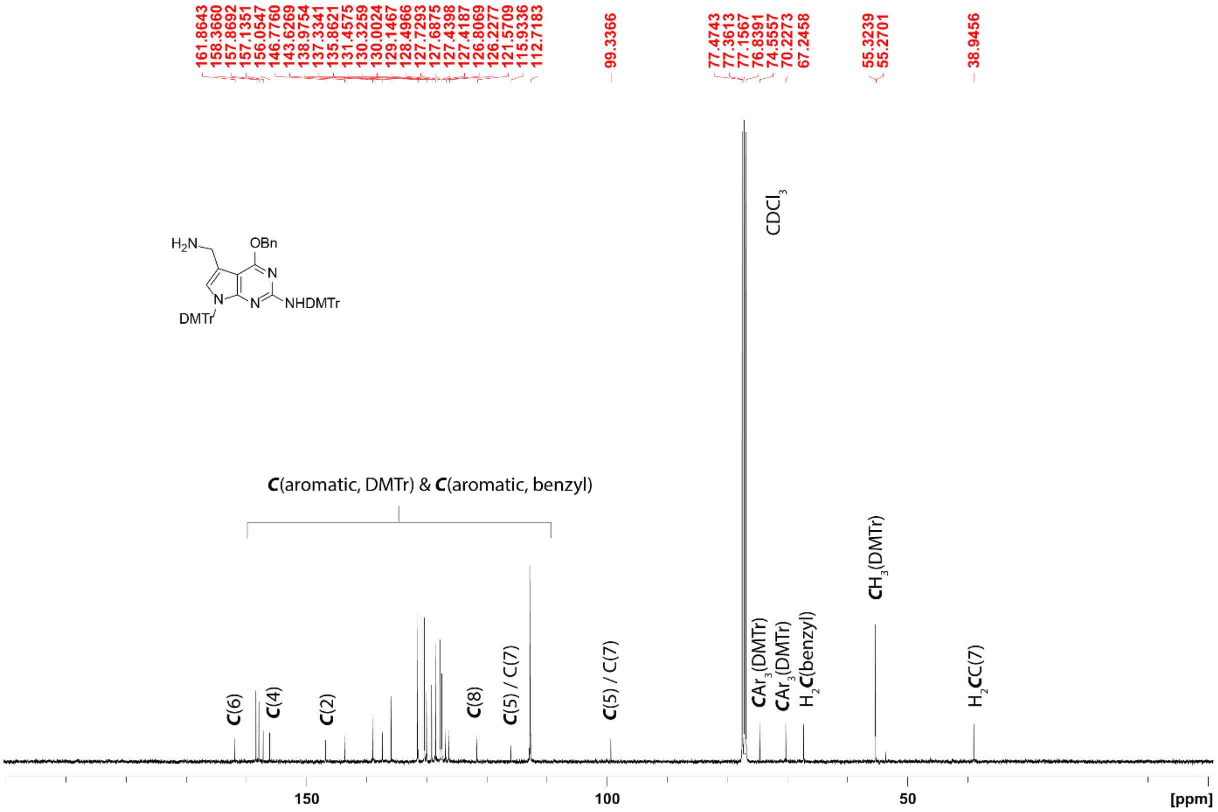

^{1}H-NMR (400 MHz, DMSO-d_6) of compound **2b**:

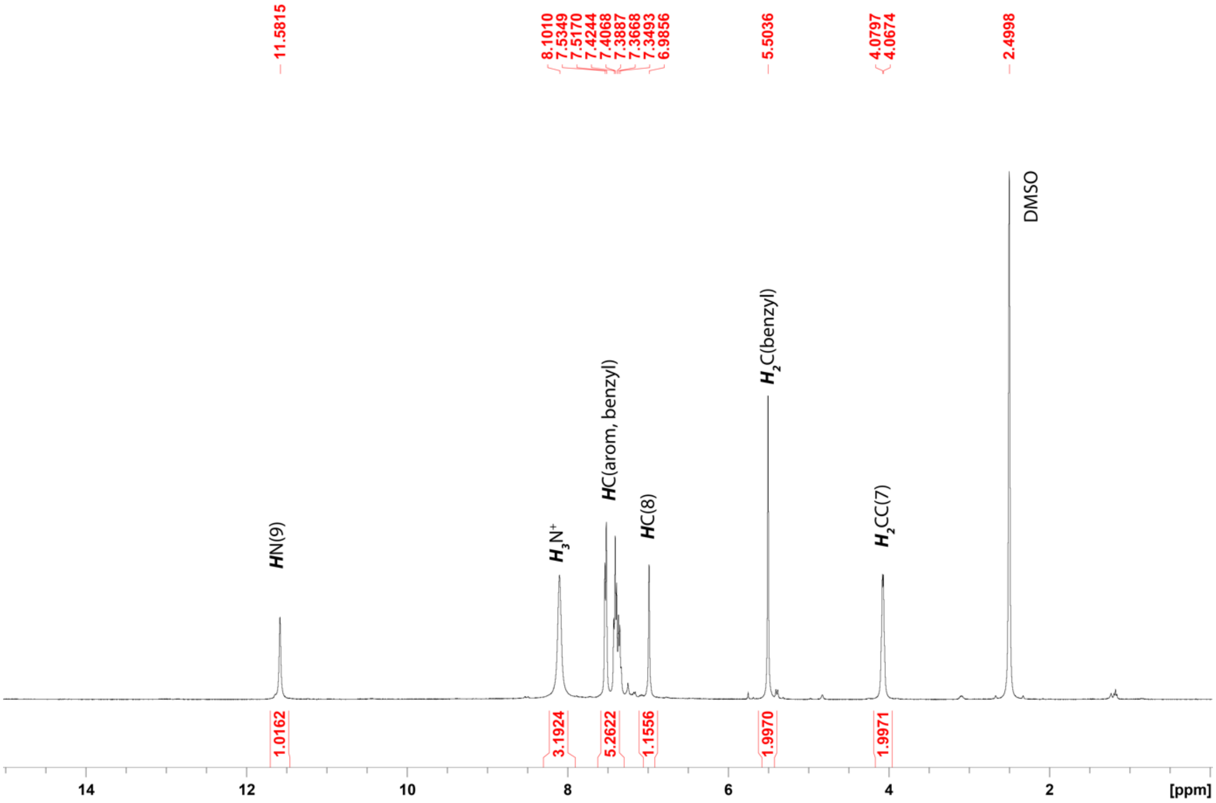

^{13}C-NMR (101 MHz, DMSO-d_6) of compound **2b**:

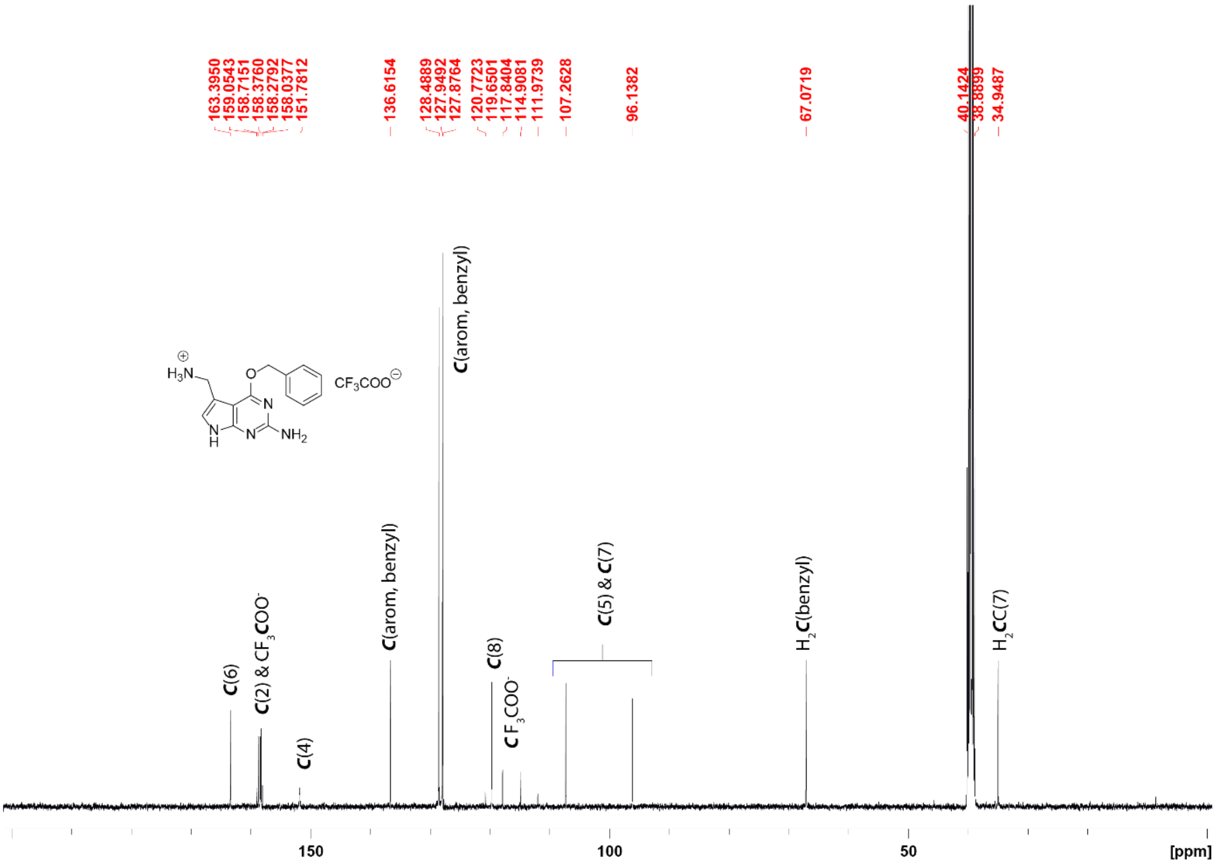

^{1}H-^{13}C HMBC NMR (400 MHz, DMSO-d_6) of compound **2b**:

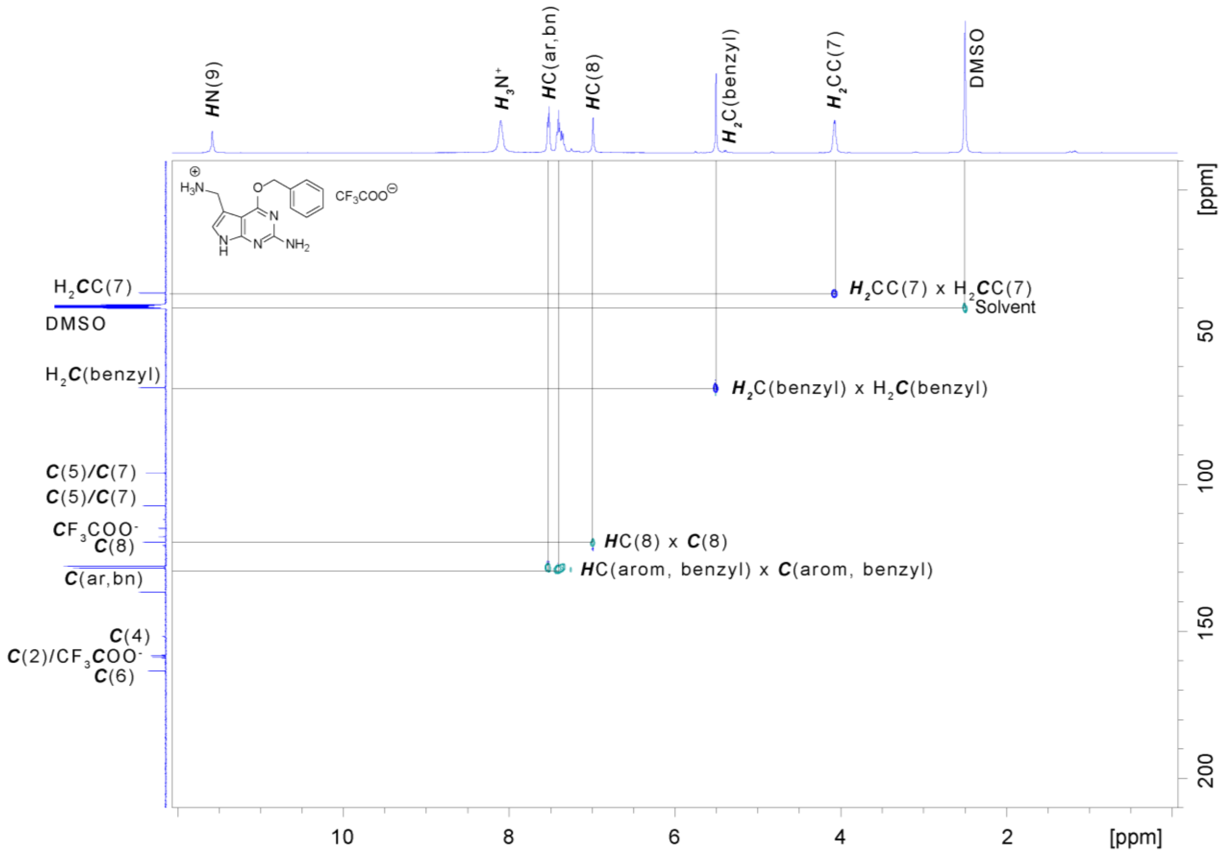

^{1}H-^{13}C HMBC NMR (400 MHz, DMSO-d_6) of compound **2b**:

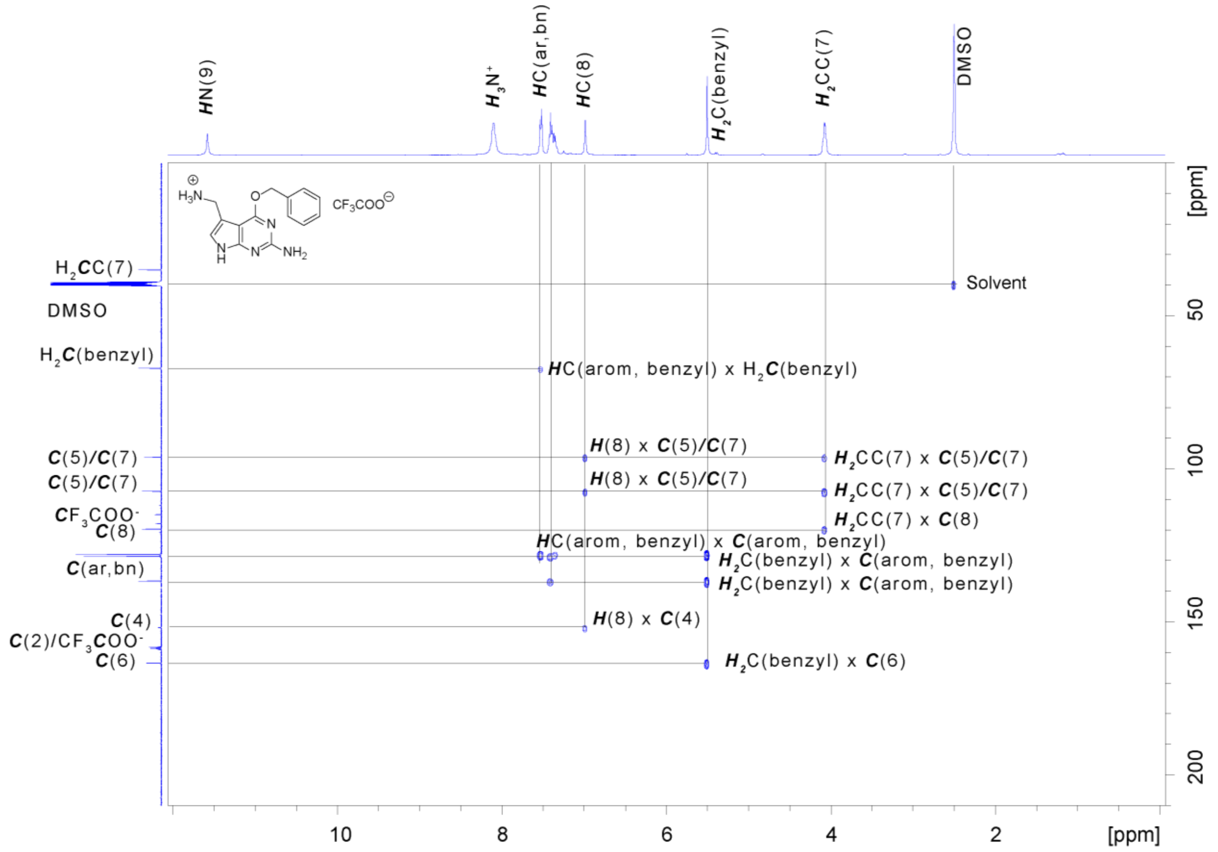

Supplement: File 1 — Synthetic procedures for compounds 1a–6a, and 1b–6b, and 1H and 13C NMR spectra of all compounds. 1H,13C HSQC and 1H,13C HMBC spectra of all final products 1, 1a, 1b, 2, 2a, and 2b. [file Beilstein_J_Org_Chem-17-2295-s001.pdf]
